# Paediatric Hypertension in Africa: A Systematic Review and Meta-Analysis

**DOI:** 10.1016/j.eclinm.2021.101229

**Published:** 2021-12-06

**Authors:** Simone H. Crouch, Larske M. Soepnel, Andrea Kolkenbeck-Ruh, Innocent Maposa, Sanushka Naidoo, Justine Davies, Shane A. Norris, Lisa J. Ware

**Affiliations:** a SAMRC/Wits Developmental Pathways for Health Research Unit, Faculty of Health Sciences, University of the Witwatersrand, Johannesburg, South Africa.; b Julius Global Health, Julius Center for Health Sciences and Primary Care, University Medical Center Utrecht, Utrecht University, Utrecht, The Netherlands.; c Division of Epidemiology and Biostatistics, School of Public Health, Faculty of Health Sciences, University of Witwatersrand, Johannesburg, South Africa School of Public Health; d Institute of Applied Health Research, University of Birmingham, Birmingham, United Kingdom; e School of Health and Human Development, University of Southampton, Southampton, United Kingdom.; f DSI-NRF Centre of Excellence in Human Development, University of the Witwatersrand, Johannesburg, South Africa.

**Keywords:** Paediatric, Hypertension, Blood pressure, Africa, Child and adolescent, BP, Blood pressure, BMI, Body mass index, CVD, Cardiovascular disease, GDP, Gross domestic product, LMIC, Low- and middle-income countires, NCD, Non-communicable diseases, UN, United Nations, WHO, World Health Organisation

## Abstract

**Background:**

The burden of cardiovascular disease (CVD) and hypertension is rapidly increasing in low- and middle-income countries. This is evident not only in adults, but also in children. Recent estimates of prevalence in children are lacking, particularly in Africa. As such, we conducted a systematic review and meta-analysis to provide updated estimates of paediatric hypertension in Africa.

**Methods:**

We searched PubMed and EBSCO to identify articles published from January 2017 to November 2020. Studies were assessed for quality. We combined results for meta-analyses using a random effects model (Freeman-Tukey arcsine transformation). Heterogeneity was quantified using the I^2^ statistic.

**Findings:**

In the narrative synthesis of 53 studies, publication bias was low for 28, moderate for 24, and high for one study. Hypertension prevalence ranged substantially (0·2%-38·9%). Meta-analysis included 41 studies resulting in data on 52918 participants aged 3 to 19 years from ten countries. The pooled prevalence for hypertension (systolic/diastolic BP≥95th percentile) was 7·45% (95%CI 5·30-9·92, I^2^=98.96%), elevated blood pressure (BP, systolic/diastolic BP≥90th percentile and <95th percentile) 11·38% (95%CI 7·94-15·33, I^2^=98.97%) and combined hypertension/elevated BP 21·74% (95%CI 15·5-28·69, I^2^=99.48%). Participants categorized as overweight/with obesity had a higher prevalence of hypertension (18·5% [95%CI 10·2-28·5]) than those categorized as underweight/normal (1·0% [95%CI 0·1-2·6], 4·8% [95%CI 2·9-7·1], p<0·001). There were significant differences in hypertension prevalence when comparing BP measurement methods and classification guidelines.

**Interpretation:**

Compared to a previous systematic review conducted in 2017, this study suggests a continued increase in prevalence of paediatric hypertension in Africa, and highlights the potential role of increasing overweight/obesity.

**Funding:**

This research was funded in part by the Wellcome Trust [Grant number:214082/Z/18/Z]. LJW and SAN are supported by the DSI-NRF Centre of Human Development at the University of the Witwatersrand.

**Research in context**

**Evidence before this study**

Mounting evidence has suggested that both hypertension and certain risk factors for its development, such as obesity, occur early in childhood. Prior to this review only one systematic review and meta-analysis focusing on paediatric hypertension specifically in Africa could be found, spanning 21 years (1996-2017) and reporting prevalence of paediatric hypertension in Africa to be 5·5%. Given the rapid increase in exposure to risk factors for hypertension in children, an update of the previous review is needed.

**Added value of this study**

We searched PubMed and EBSCO to identify articles published from January 2017-November 2020. Studies were assessed for quality and risk of bias. We identified and included 53 and 41 studies in the narrative review and meta-analysis, respectively, suggesting an increase in relevant publications from the previous review, which included 25 studies in the meta-analysis spanning 21 years. The pooled prevalence for hypertension was 7·5%, a 36% increase from the previous review and meta-analysis.

**Implications of all the available evidence**

Despite the call made in the previous review for measures to reduce paediatric hypertension, it is clear hypertension levels have continued to rise. This highlights the urgency for implementation of prevention strategies across Africa. Given the strong association between blood pressure and BMI, strategies should include evidence-based primary prevention programmes, enhanced development and availability of contextually-relevant paediatric guidelines, and increasing the funding and resources for awareness, detection and management of paediatric hypertension.

Alt-text: Unlabelled box

## 1 Introduction

The prevalence of non-communicable diseases (NCDs) remains a growing concern globally, with a burgeoning NCD burden in low- and middle-income countries (LMICs) [1]. According to the World Health Organization (WHO), cardiovascular disease (CVD) alone accounts for approximately 17.9 million NCD deaths annually [2], 75% of which occurred in LMICs [2]. This increase in NCDs seen in LMICs, including in Africa, may result from “rapid, unplanned and unmanaged” urbanisation [3], often associated with an increase in CVD risk factors such as dietary changes, increasingly sedentary lifestyles, increasing obesity, tobacco use and exposure to air pollutants [3], [4], [5], [6]. The risks are not only evident in adults, but also in children. Obesity in children and adolescents in Southern Africa has shown the largest proportional increase globally with a staggering 400% increase per decade [7]. Obesity is, in turn, associated with elevated blood pressure (BP) and hypertension [8], a significant contributor to the development of CVD [9]. A previous systematic review evaluating paediatric hypertension in Africa between 1996 and 2017 (21 years) included only 51 studies of which 25 were included in the meta-analysis [10]. Due to the rapid increase in risk factors, the previous review's finding that obesity is significantly associated with hypertension, and the growing focus on the importance of paediatric hypertension, an update of this previous review is urgently needed.

Hypertension is the leading risk factor not only for CVD, but for the burden of disease globally [11]. The prevalence of hypertension in children and adolescence is of great concern since elevated BP in childhood and adolescence tracks into adulthood in the majority of cases [12,13]. One study found that in adults presenting with hypertension, around half had elevated BP in childhood. Additionally, elevated BP in childhood has been shown to predict increased adult cardiovascular disease and mortality, including coronary heart disease and stroke [14,15]. Furthermore, the risk factors for paediatric elevated BP, such as obesity, may also track into adulthood, highlighting the importance of interventions at an early age [16]. Despite the clear importance of evaluating childhood and adolescent BP there are no paediatric BP guidelines for the African region at present.

The aim of this systematic review and meta-analysis is to present a detailed, updated review of the prevalence of hypertension in children and adolescents in Africa, evaluating the availability of information and the impact of covariates such as obesity, age, and sex. Furthermore, we aimed to examine the methods and guidelines used to determine hypertension in child and adolescent populations in Africa

## 2 Methods

This systematic review and meta-analyses was conducted in accordance with the PRISMA guidelines.

### 2.1 Search Strategy

This review aims to serve as an update of a previous review evaluating paediatric hypertension in Africa between 1 January 1996 and 2 February 2016, and as such will closely mimic their search strategy [10]. The search for articles was conducted in November 2020 according to the PICO (Participants, Intervention, Comparator, Outcomes) model of formulating a clinical question in the healthcare setting. Restrictions to articles were based on age (between 1-19 years), study population (African countries), date of publication (from 1 January 2017 until 30 November 2020), and language (published in English). The following databases were used in the search: PubMed, EBSCO host (including Scopus, African Journals, academic search complete and Medline). A sample of the EBSCO search strategy is available in supplementary file 1. References of identified articles were also screened for additional relevant articles that met the inclusion criteria.

### 2.2 Screening and selection

The results were screened for duplicates which were removed, followed by title and abstract screening; screening was completed by one researcher (SHC), with 10% of titles and abstracts additionally screened by a second member of the research team to check agreement. Full texts of eligible articles were then accessed and divided among three authors (SHC, LMS, AKR) for full text screening, and 10% of full-text articles were screened by a second member of the research team to check agreement. If a full text was unavailable, the authors were contacted to gain access to the article. Full-text articles meeting inclusion criteria for the meta-analyses were double screened.

### 2.3 Study Selection

Randomised control trials, cohort studies, case studies, longitudinal and cross-sectional studies reporting prevalence of elevated blood pressure (BP) (prehypertension), hypertension (systolic and/or diastolic), or combined elevated BP and hypertension in children aged 1-19 years, were included. Letters, reviews, commentaries and editorials as well as animal and genetic studies, studies not written in English, studies among populations of African origin residing outside of Africa, studies selecting participants on the basis of presence of hypertension, and studies not differentiating between adult and child/adolescent data were excluded.

For the meta-analysis, studies were additionally excluded if no raw prevalence data could reliably be extracted (for example, if only a percentage was provided), if no aggregate systolic and diastolic hypertension prevalence data were available, in case of high risk of bias (described below), and/or if participants were selected from within a specific disease. Additionally, if two studies from the same database were identified, the study with the lowest risk of bias and largest sample size was included in the meta-analysis.

Any disagreements in full text screening and selection were resolved through discussion among three authors (SHC, LMS, AKR) until consensus was reached.

### 2.4 Risk of bias assessment

We used a risk of bias tool specific to prevalence studies developed by Hoy et al to assess risk of bias [17]. Each study was assessed by SHC, LMS and AKR according to the tool's criteria, resulting in a summary score per paper that was categorised as follows: 0-5 high risk of bias; 6-7: moderate risk of bias; 8-10: low risk of bias [18].

### 2.5 Data extraction

Relevant data from each individual paper was extracted using a predefined, standardized data extraction form (Supplementary table 1). Where relevant information for inclusion in the meta-analysis was not available, we contacted the relevant study's corresponding author, allowing two weeks for a response.

Relevant data included author name, year of publication, year of data collection, country, geographical setting (urban, peri-urban or rural), classification used to determine hypertension status, age range of participants, mean age of participants, participant sex, participant body mass index (BMI), total sample size, prevalence (n and % - if one was not available data n or % was calculated from the other if possible) of hypertension (systolic and/or diastolic BP ≥95% percentile), prevalence (n and %) of elevated BP (systolic and/or diastolic BP ≥90% and <95% percentile), and prevalence (n and %) of combined hypertension and elevated BP (2017 AAP guidelines) [19]. We additionally determined the African region (Eastern, Western, Central, Southern, Northern) according to the United Nations (UN) classification [20] and ascertained country gross domestic product per capita (GDP) according to the World Bank [21].

### 2.6 Data analysis

We performed meta-analysis on the subset of papers meeting the selection criteria outlined above. Using STATA 13 (StataCorp, 2013, College Station, USA), we pooled individual study estimates using a random effects model for meta-analysis following the Freeman-Tukey arcsine transformation to stabilize the variance [22].

Heterogeneity was quantified using the I^2^ statistic [23]. For the hypertension outcome, subgroup analysis using ANOVA was performed in case of significant heterogeneity, comparing the following *a priori* determined variables: African region, geographical setting (urban or rural), timing of data collection before or after 2015, age, sex, BMI category (underweight, normal, overweight/with obesity), BP measurement method (automatic/oscillometric vs manual/auscultatory), number of occasions of BP measurement, standards used for categorisation of hypertension (for example, the AAP 2004 “Fourth Report; [24] the AAP 2017 guidelines [19]) sample size, and risk of bias score. A p-value of <0.05 was set to indicate a significant difference between subgroups. Funnel plots and the Egger test p-value were used to assess the presence of publication bias, considered to be present at a p-value of <0.1 in analyses with at least five studies included. [25]

Additionally, meta-regression analysis was performed to further explore heterogeneity with respect to prevalence of hypertension. Following univariate regression analysis, we performed multivariable regression analysis across three models, namely: Model 1 (M1): country GDP and mean BMI; Model 2 (M2): country GDP, mean BMI and age; Model 3 (M3): country GDP, mean BMI, Age, automatic or manual BP, number of BP measurements.

## 3 Ethical considerations

As a meta-analysis without original data this study was exempt from ethical approval. To our knowledge all includes studies obtained ethical approval from their respective institutions.

## 4 Role of Funding

The funders of this study had no role in the study design, data collection, data analysis, data interpretation, or the writing of the article. SHC, LMS and AKR had full access to all the data in the study. All authors had access to data extraction sheet and outputs as well as final responsibility for the decision to submit for publication.

## 5 Results

**Figure 1:**
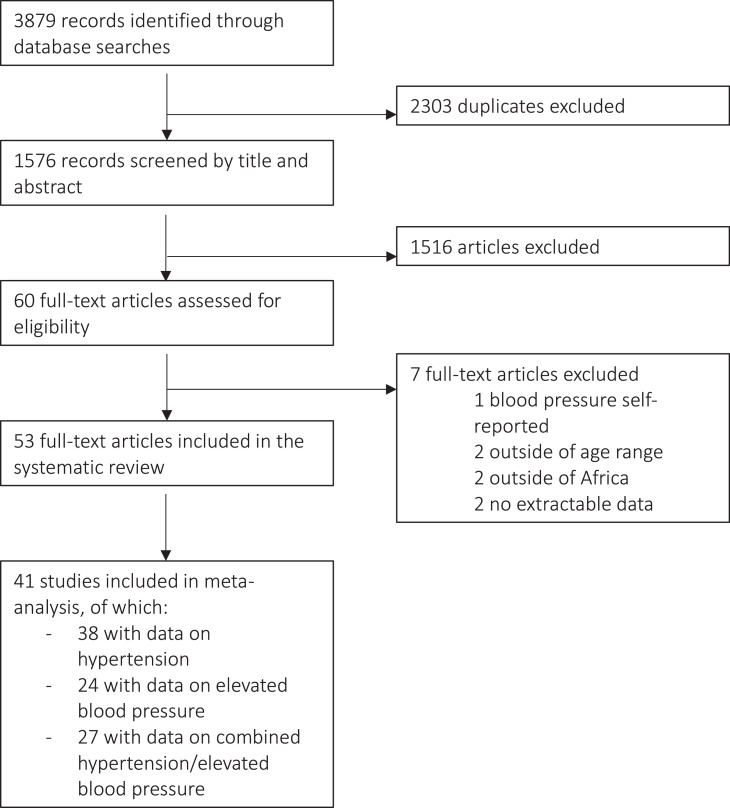
Flow diagram of the study selection.

Search results were screened for duplicates by title and abstract screening; 1516 of the 1576 articles were excluded as they did not focus on or report paediatric hypertension or were conducted outside of Africa. Of 60 articles identified from the title and abstract screening, 53 presented data on hypertension prevalence specifically for African children between 1-19 years age range. Reasons for the exclusion of the remaining 7 articles were: Blood pressure (BP) was self-reported and not measured (n=1) (Letamo et al) [26], the study sample spanned outside the desirable age range in which no age specific data could be extracted (n=2) (Bhimma et al [27], Mokgwathi et al [28]), the study was not conducted within an African population residing in Africa (n=2) (South et al [29], South et al [30]) and no extractable information regarding hypertension could be extracted from the results (n=2) (Muyumba et al [31], Mphekgwana et al [32]) (Figure 1).

Information regarding prevalence of paediatric elevated BP, hypertension and/or combined elevated BP and hypertension was obtained for 58591 participants from ten African countries (representing 18.5% of African countries); Algeria, Cameroon, Egypt, Gambia, Ghana, Nigeria, Seychelles, South Africa, Tanzania, and Uganda. South Africa and Nigeria were represented by 16 studies each, whereas the other countries had between 1-5 studies each. Of all included studies, 51% (n=27) reported both hypertension and elevated BP prevalence, this was followed by 34% (n=18) only reporting on hypertension prevalence, while the remaining 15% reported on either elevated BP (n=4) or combined hypertension and elevated BP (n=4).

Of the studies included in the systematic review, 27 were categorized as low risk of bias, 25 as moderate risk, and one as high risk of bias (Supplementary table 3). External validity (study population, sampling frame, and participant selection) were the main individual items presenting higher risk of bias scores.

An overview of the included studies, subdivided by African regions, can be found in Table 1. In the majority of studies (n=33), hypertension and elevated BP percentiles were calculated using the standards from the “Fourth report on the diagnosis, evaluation and treatment of high BP in children and adolescents” (Fourth Report, AAP 2004) [24]. Five studies did not specify which standards they used to determine their age, sex, and height adjusted percentiles, while one study reported using only SBP and/or DBP ≥130/85 as their hypertension classification [33].

**Table 1:** Prevalence of hypertension and elevated blood pressure in African children and adolescents

**Author**	**Country / Setting**	**Year data collected**	**Sample size (n)**	**Age range yrs@(mean)**	**(n) males / (n) females**	**Hypertension criteria used**	**% Hypertension**			% Elevated BP / Combined high BP*			**% Obese / Overweight + obese**^†^
							**All**	**M**	**F**	**All**	**M**	**F**	**All**
**Northern Africa**													

Benmohammed et al 2018 [70]	Algeria / Urban	2007	1100	12-18 (15·1)	537 / 563	4^th^ report 2004	12·4			13			4·6
Bouhenni et al 2017 [90]	Algeria / Both	2014	577	10-19 (15·2)	261 / 316	4^th^ report 2004	4·3			14·7			
El-Koofy et al 2020 [34]	Egypt	2016	72	3-14 (8·7)	40 / 32	4^th^ report 2004	38·9						55·6
Elseifi et al 2020 [35]	Egypt	2017-2018	224	12-14 (13·0)	115 / 109	2017 AAP				Systolic: 3·6 Diastolic: 2·7			18·8
^a^Hassan et al 2019 [37]	Egypt	2013-2016	200	12-18 (16)	0 / 200	4^th^ report 2004				50·5			37·5^†^
^a^Hassana et al 2019 [36]			77		35 /42					40·3	42·9	38·8	100
Sherif et al 2019 [38]	Egypt / Urban	2016-2017	110	4-18	38 / 72	Percentiles, undefined				Systolic: 9·1 Diastolic: 10			3·6

**Eastern Africa**													

Katamba et al 2020 [39]	Uganda / Peri-urban	2018	616	12-19 (15·6)	212 / 404	4^th^ report 2004	3·1			7·1	8·5	6·4	3·4
Leyvraz et al 2018 [40]	Seychelles	1998-2006	4519	5-6 (5·5)	2324 / 2195	4^th^ report 2004	10·2	9·1	11·4				5·9
Lule et al 2019 [91]	Uganda / Rural	2014-2016	1119	(10·2)	583 / 536	4^th^ report 2004	8·4			10·5			
Muhihi et al 2018 [41]	Tanzania / Urban		446	6-17 (11·1)	209 / 237	4^th^ report 2004	10·8			4·9			5·2
Nakiriba et al 2018 [42]	Uganda / Peri-urban		688	12-19 (15·4)	0 / 688	Percentiles, undefined	11·6						30·5
Nsanya et al 2019 [72]	Tanzania, Uganda / Urban	2015	827	12-17	410 / 417	4^th^ report 2004	Total: 15·0 12-14 yrs: 15 15-17 yrs: 15	12-14 yrs: 6 15-17 yrs: 16	12-14 yrs: 21 15-17 yrs: 14	Total: 22·0 12-14 yrs: 16 15-17 yrs: 23	12-14 yrs: 17 15-17 yrs: 28	12-14 yrs: 16 15-17 yrs: 17	
Nyangasa et al 2019 [43]	Tanzania -@Zanzibar / Rural	2013	165	4·9-18 (12)	85 / 80	4^th^ report 2004	9·7			15·1			5·5^†^

**Southern Africa**													

^b^Gerber et al 2018 [44]	RSA / Urban	2015-2016	801	8-13 (9·5)	402 / 399	Neuhauser et al 2017	32·6	31·8	33·3	8·4	7·5	9·3	5·1
^b^Nqwenso et al 2020 [73]			842		433 / 409	Neuhauser et al 2017 (only if both SBP + DBP	13·5			7			
*Combined high BP; † Overweight + Obese; Studies with same superscript ^(a,b,c)^ made use of same dataset, however made use of either different classifications or study outcomes.													
Gomwe et al 2019 [75]	RSA		876	9-14 (11)	356 / 520	4^th^ report 2004	Systolic: 5·3 Diastolic: 2·6	Systolic: 4·8 Diastolic: 2·5	Systolic: 5·6 Diastolic: 2·7	Systolic: 18·4 Diastolic: 14·7	Systolic: 16·6 Diastolic: 11·8	Systolic: 19·6 Diastolic: 16·7	
Sekokotla et al 2017 [45]	RSA/ Urban		371	13-18 (15·2)	116 / 255	Percentiles, undefined					32·6*		40·2^†^
^c^Matjuda et al 2020 [92]	RSA / Both	2018	306	6-9 (8)	135 / 171	2017 AAP	10·5	8·1	12·2	32·3	39·3	25·7	
^c^Matjuda et al 2020 [46]										42·2*			19·3^†^
Chungag et al 2019 [47]	RSA / Urban	2016	540	10-14 (11·9)	250 / 290	4^th^ report 2004	20·7	15·6	26·2	12·2	11·2	15·5	14
Mphekgwana et al 2019 [32]	RSA / Rural	200	1811	5-16	934 / 877	4^th^ report 2004	1·3						
Sekgala et al 2017 [93]	RSA / Rural	1999-2003	9002	6-17	4678 / 4324	4^th^ report 2004	4·4			5·3			
^d^Sebati et al 2020 [48]	RSA		1665	5-15 (9·9)	846 / 819	Joint National Committee 7^th^ report, 2003	4·3	Total: 3·3 5-7 yrs: 3·6 8-10 yrs: 7·4 11-15 yrs: 21·3	Total: 5·3 5-7 yrs: 1·4 8-10 yrs: 14·1@11-15 yrs: 33				10·2^†^
^d^Nkwana et al 2019 [94]						Percentiles, unclear	14·4						
Mokwatsi et al 2017 [94]	RSA/ Urban	2015	81	6-8 (7·3)	81 / 0	Percentiles, undefined	6·2			12·3			
Masocha et al 2020 [50]	RSA	2011-2013	186	14-16 (14·9)	81 / 105	NCEP/ATP III criteria, 2007				5	5	5	13^†^
Raphadu et al 2020 [51]	RSA/ Both		218	13-19 (17)	97 / 121	4^th^ report 2004	17·1			27·3			5·5
Houle et al 2019 [95]	RSA/ Rural	2012-2014	1536	7-11 (9·3)		4^th^ report 2004	4·2			4·9			13·2
Negash et al 2017 [52]	RSA/ Rural	2007-2008	1559	7-18 (13)	619 / 940	4^th^ report 2004	2·6	2·9	2·4				7·3

**Western Africa**													
Schoenbuchner et al 2018 [74]	Gambia / Rural	2012-2015	2773	10-14·9: (12·5)@15-19·9: (17·1)	1405 / 1368	4^th^ report 2004		10-14 yrs: 9·5 15-19·9 yrs: 2	10-14·9 yrs: 8 15-19·9 yrs: 4				
Jobe et al 2017 [96]	Gambia / Rural	2012-2014	3637	5-17·9	1907 / 1730	4^th^ report 2004	8·2						
Azupogo et al 2020 [53]	Ghana / Both	2014	1727	15-19 (16·9)	870 / 857	4^th^ report 2004	0·2			20·4			7·1^†^
Amponsem-Boateng et al 2019 [54]	Ghana / Urban	2018-2019	699	<15@15-17		Joint National Committee 7^th^ report, 2003	Total: 3·3@<15 yrs: 3·9@15-17 yrs: 3·2			Total: 32·6 <15 yrs: 21·9 15-17 yrs: 35			7·4, 34·4^†^
Alicke et al 2017 [55]	Ghana / Urban	2015	188	14·5-15·5	94 / 94	4^th^ report 2004	9	10	7				7·0^†^

Ibrahim et al 2019 [56]	Nigeria / Both	2014-2015	1745	6-12 (8·8)	873 / 872	4^th^ report 2004	3						0·6
Okpokowuruk et al 2017 [97]	Nigeria / P-Urban		200	3-17 (12·4)	72 / 128	4^th^ report 2004	3·5			2·5			
Abu et al 2020 [57]	Nigeria	2015	420	10-19 (14)	179 / 241	4^th^ report 2004	6·9			8·8			3·3
Abiodun et al 2019 [58]	Nigeria	2014-2017	6980	15-19 (16·5)	3059 / 3921	4^th^ report 2004	25·3	26·1	14·9	25·1	31·3	20·4	9·6
Emmanuel et al 2017 [59]	Nigeria		416	10-19 (14·8)	208 / 208	4^th^ report 2004	10·1	5·8	14·4				25^†^
Ezeudu et al 2018 [60]	Nigeria / Urban	2013-2014	984	10-19 (14·6)	470 / 514	4^th^ report 2004	6·3	5·4	7·3	5·0	4·2	5·8	2·5
Amadi et al 2019 [61]	Nigeria	2017	491	@6-17@	219 / 272	4^th^ report 2004	Total: 9·4 6-12 yrs: 6·5 13-17 yrs: 14·2	5·9	12·1				15·0
Omisore et al 2018 [62]	Nigeria / Both	2012	1000	10-16 (13·7)	510 / 490	4^th^ report 2004	4·1						2·9
Adeomi et al 2019 [63]	Nigeria / Urban		313	10-19 (14·4)	130 / 183	4^th^ report 2004				32·9*	26·2*	37·7*	10·2^†^
Ukoh et al 2020 [64]	Nigeria / Urban	2015-2016	2401	10-19 (15·1)	1196 / 1205	4^th^ report 2004	4·6	3·8	5·4				1·3
Musa et al 2020 [33]	Nigeria / Rural	2019	197	11-18 (14·6)	97 / 100	SBP/DBP ≥130/85	Systolic: 5·1 Diastolic: 12·2	Systolic: 3·1 Diastolic: 7·2	Systolic: 7 Diastolic: 17				
Isezuo et al 2018 [65]	Nigeria	2014-2015	800	10-18 (14·5)	424 / 376	4^th^ report 2004	Total: 3·1 10-13 yrs: 0·4 14-16 yrs: 3·9 16-18 yrs: 6·3	3·3	2·9	Total: 7·5 10-13 yrs: 2·9 14-16 yrs: 8·8 16-18 yrs: 12·6	7·3	7·7	0·3
Yilgwan et al 2017 [66]	Nigeria / Urban		241	6-12 (9·2)	104 / 137	Joint National Committee 7^th^ report, 2003	9·1	10·6	8				13·7
Akinbodewa et al 2020 [98]	Nigeria / Rural		114	3-9 (5·6)@10-17 (12·9)	55/59	4^th^ report 2004	Total: 7 3-9 yrs: 1·6 10-17 yrs: 13·2	1·8	11·9	Total: 12·3 3-9 yrs: 11·5 10-17 yrs: 13·2	9·1	15·3	
Sadoh et al 2017 [67]	Nigeria / Urban	2011-2012	1466	5-15 (9)	814 / 652	4^th^ report 2004	2·7	1·8	3·6	3·1	2·2	3·9	5·7
Wariri et al 2018 [71]	Nigeria / Rural	2015	367	10-18 (14·9)	191 / 176	4^th^ report 2004	5·7	4·2	7·4	10·6	9·1	11·4	

**Central Africa**													

Chedjou-Nono et al 2017 [68]	Cameroon	2013-2014	76	2-17 (9·9)		4^th^ report 2004	Obese: 25·0 Control: 0			Obese: 19·4 Control: 5·3			50
Chelo et al 2019 [69]	Cameroon / Both	2017-2018	822	5-17 (9·0)	353 / 469	2017 AAP	1·6			8·2			0·6

*Combined high BP; †Overweight + Obese; Studies with same superscript ^(a,b,c)^ made use of same dataset but made use of either different classifications or study outcomes.

**Table 2:** Subgroup analyses performed for meta-analysis of hypertension prevalence

**Subgroup**	N studies	Number of participants	Prevalence (95% CI)	I^2^ (%)	p-values		
					Heterogeneity	Heterogeneity between groups	Egger test
**Africa region** Western Northern Central Southern Eastern Total	18 3 1 9 7 38	23,876 1749 822 17,207 8380 52,034	6·0 (2·8-10·2) 15·2 (5·4-28·8) 1·6 (0·9-2·7) 7·9 (4·0-12·9) 9·5 (7·1-12·3) 7·5 (5·3-9·9)	99·3 - - 98·9 92·1 99·0	<0·001 - - <0·001 <0·001 <0·001	<0·001*	0·029* - - 0·263 0·863 0·452
**Geographical setting** Urban Rural Total	9 11 20	8805 21,069 29,874	9·2 (4·6-15·1) 5·3 (3·8-7·0) 7·0 (5·0-9·3)	98·6 95·4 97·8	<0·001 <0·001 <0·001	0·129	0·465 0·700 0·226
**Timing of data collection** Only after 2015 Before 2015 Total	14 17 31	8631 39,633 48,264	10·0 (5·7-15·4) 5·6 (2·9-9·1) 7·4 (5·0-10·2)	98·1 99·4 99·1	<0·001 <0·001 <0·001	0·108	0·410 0·140 0·484
**Age group** Over 13 Under 13 Total	8 13 21	11,673 12,673 24,346	6·8 (0·8-17·5) 6·9 (3·8-10·8) 6·8 (3·5-11·1)	99·6 97·8 99·2	<0·001 <0·001 <0·001	0·953	0·050* 0·847 0·048*
**Sex** Male Female Total	16 16 32	10,791 11,488 22,279	8·2 (3·5-14·7) 10·8 (7·4-14·7) 9·5 (6·4-13·0)	99·0 97·1 98·5	<0·001 <0·001 <0·001	0·478	0·133 0·482 0·095*
**BMI Category** Underweight Normal Overweight/Obese Total	4 9 9 22	1809 6885 823 9517	1·0 (0·1-2·6) 4·8 (2·9-7·1) 18·5 (10·2-28·5) 7·5 (5·0-10·3)	63·5 93·4 90·5 95·0	<0·001 <0·001 <0·001 <0·001	<0·001*	- 0·449 0·302 0·009*
**BP methodology** Automatic (oscillometric) Manual (auscultation) Total	21 11 32	32,806 8230 41,036	8·2 (6·0-10·6) 4·6 (3·3-6·0) 6·9 (5·3-8·6)	98·1 87·9 97·5	<0·001 <0·001 <0·001	0·007*	0·171 0·094* 0·275
**Number of measurement occasions to define HTN** Single Multiple Total	26 10 36	35,832 8802 44,634	8·0 (5·9-10·5) 4·8 (3·3-6·5) 7·0 (5·4-8·8)	98·3 91·0 97·8	<0·001 <0·001 <0·001	0·018*	0·122 0·494 0·150
**Standards used for classification of HTN** Fourth Report, AAP 2004 Clinical Practice, AAP 2017 Other/unclear Total	30 2 6 38	46,731 1128 4175 52,034	7·2 (4·9-9·9) 3·2 (2·3-4·4) 9·8 (2·0-20·1) 7·5 (5·3-9·9)	99·1 - 98·8 99·0	<0·001 - <0·001 <0·001	0·003*	0·367 - 0·849 0·477
**Sample size** >699 (median) <699 Total	20 18 38	45,407 6627 52,034	6·3 (3·6-9·7) 8·8 (6·4-11·5) 7·5 (5·3-9·9)	99·4 92·4 99·0	<0·001 <0·001 <0·001	0·206	0·208 0·337 0·477
**Risk of bias score** Moderate Low Total	15 23 38	15,476 36,558 52,034	11·5 (6·3-18·0) 5·3 (3·9-6·9) 7·5 (5·4-9·9)	99·1 97·3 99·0	<0·001 <0·001 <0·001	0·021*	0·129 0·973 0·477

BMI: Body mass index; BP: Blood pressure; HTN: hypertension; * indicates statistical significance (p<0.05)

The overall prevalence of hypertension, elevated BP and combined hypertension/elevated BP ranged from 0·2% to 38·9%; 2·5% to 40·3%; and 32·9% to 50·5% respectively. In males, hypertension ranged from 1·8% to 31·8%, while in females, hypertension prevalence was between 2·4% to 33·3%. Elevated BP ranged from 2·2% to 39·3% and from 3·9% to 25·7% in males and females, respectively. The prevalence of both hypertension and elevated BP differed within the various African regions, with Northern Africa showing the highest prevalence (range: 4·3% - 38·9%) and Eastern Africa showing the lowest prevalence (range: 3·1% - 15%)·

Of the 53 studies, 37 reported obesity and/or overweight prevalence in their respective samples [34], [35], [36], [37], [38], [39], [40], [41], [42], [43], [44], [45], [46], [47], [48], [49], [50], [51], [52], [53], [54], [55], [56], [57], [58], [59], [60], [61], [62], [63], [64], [65], [66], [67], [68], [69], [70], with the prevalence of obesity ranging from 0·3% to 50% These studies consistently found a higher prevalence of elevated BP and/or hypertension in participants with obesity, overweight, or central obesity. A case-control study where authors compared prevalence of hypertension in children with and with out obesity found that only the children in the obesity group had hypertension (25%), and this group had significantly more cases of elevated BP than the non-obesity group (19·4% vs 6·5%) (Chedjou-Nono et al [68]). Additionally, three studies found that more than 20% of children with obesity or adolescents had hypertension (Emmanuel et al [59], Adeomi et al [63], Ibrahim et al [56]). Similarly, Muhihi et al reported 17.2% of overweight and having obesity children and or adolescents had elevated BP [41]. Of note, Benmohammed et al found that boys with obesity had a significantly higher prevalence of hypertension compared to girls with obesity (36% vs 27%, p=0.002) [70]. Of the included studies that quantified the association with obesity/overweight, all but one (Alicke et al, sample size 188^55^) found a significant association, with the adjusted odds of having hypertension found to be between three to 25 times increased in children with overweight and/or obesity vs normal BMI in ages ranging from 7-18 years [52,^69^,^71^,72].

### 5.1 Meta-analysis results

Of the 53 studies included in the systematic review, in total 41 were included for meta-analysis for at least one outcome (hypertension, elevated BP, or both combined). Reasons for exclusion of the remaining 12 articles were as follows: high risk of bias (Masocha et al [50]), study from the same database included (Nqweniso et al [73], Schoenbuchner et al [74], Matjuda et al [46], Hassana et al [36], Nkwana et al [49]), lack of aggregated data for systolic/diastolic hypertension (Sherif et al [38], Elseifi et al [35], Musa et al [33], Gomwe et al [75]), and lack of extractable raw numerator or denominator data (Raphadu et al [51], Chedjou-Nono et al [68]) (Figure 1). This resulted in 38 studies, 24 studies, and 27 studies included for hypertension, elevated BP, and combined hypertension/elevated BP, respectively.

**Figure 2:**
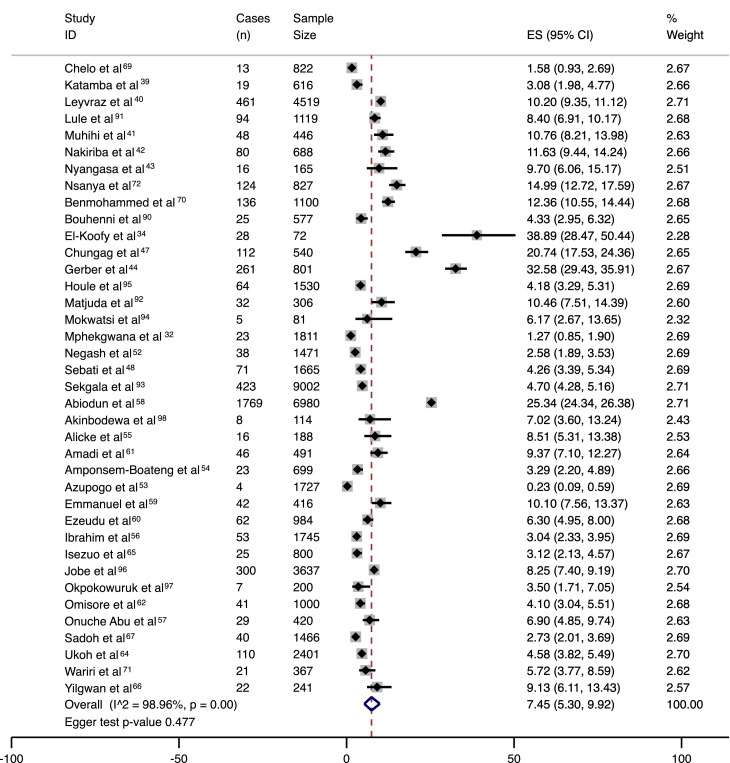
Meta-analysis results in the form of a forest plot for prevalence of hypertension with cases (n), sample size, 95% confidence intervals, estimated prevalences and percent weight per included study. ES= estimated prevalence.

**Figure 3:**
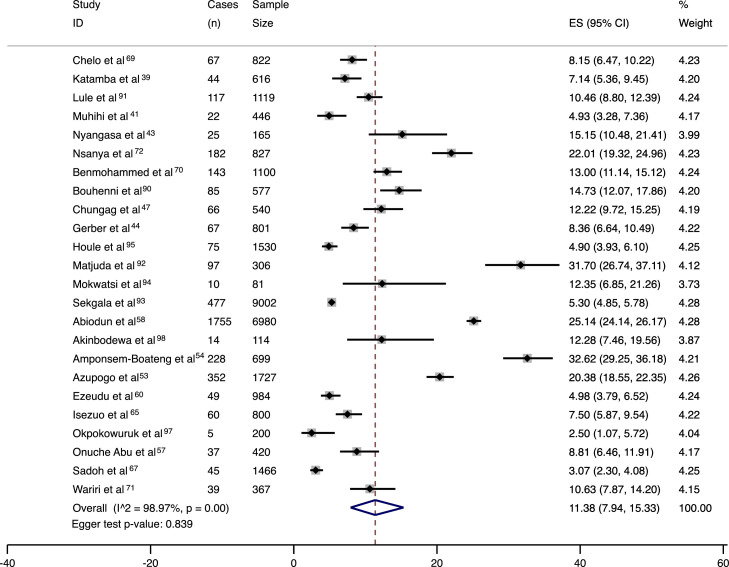
Meta-analysis results in the form of a forest plot for prevalence of elevated blood pressure with cases (n), sample size, 95% confidence intervals, estimated prevalences and percent weight per included study. ES= estimated prevalence.

**Figure 4:**
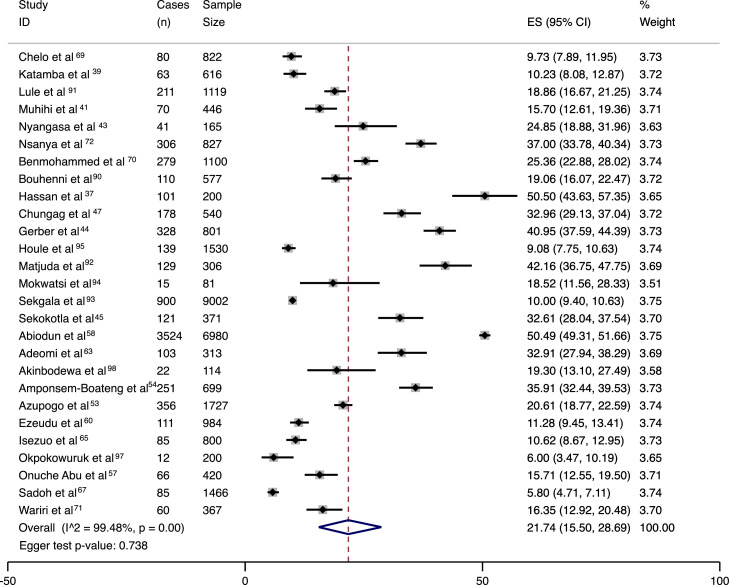
Meta-analysis results in the form of a forest plot for prevalence of combined hypertension and elevated blood pressure with cases (n), sample size, 95% confidence intervals, estimated prevalences and percent weight per included study. ES= estimated prevalence.

The forest plots of pooled prevalence for hypertension, elevated BP, and combined hypertension/elevated BP are shown in Figures 2, 3, and 4, respectively. None of these three meta-analyses showed significant asymmetry indicative of publication bias, as indicated by the funnel plot (supplementary figure 1) and the Egger test (Figure 2-4). The pooled prevalence for hypertension was 7·45% (CI 5·30-9·92), and for elevated BP was 11·38% (CI 7·94-15·33). The pooled prevalence for combined hypertension/elevated BP was 21·74% (CI 15·5-28·69). High between-study heterogeneity was found, with an I^2^ statistic of 98·96%, 98·97%, and 99·48% respectively for the analysis of hypertension, elevated BP, and combined hypertension/elevated BP.

In subgroup analysis, the Northern (3 studies) and Eastern African (7 studies) regions had the highest prevalence of hypertension at 15·2% (95% CI 5·4-28·8) and 9·5% (7·1-12·3), respectively. No significant difference in hypertension prevalence was found for studies conducted in urban vs rural settings, although 18 of the included studies did not report on geographical setting. Similarly, while studies with more recent timing of data collection (after 2015) showed a higher prevalence (10·0% [95%CI 5·7-15·4] vs 5·6% (95% CI 2·9-9·1)), this subgroup difference was not statistically significant.

Participants categorized as overweight or with obesity were found to have a significantly higher prevalence of hypertension (18·5% [95% CI 10·2-28·5]) than those categorized as underweight or normal BMI (1·0% [95%CI 0·1-2·6], 4·8% [95% CI 2·9-7·1], p<0·001). No significant difference was found for age or sex subgroups.

In terms of diagnostic methodology, a significantly higher prevalence of hypertension was found for studies using automated (oscillometric) BP measurement (8·2% [95%CI 6-10·6]) than those using manual auscultation (4·6% [95% CI 3·3-6·0], p=0·007). Moreover, studies measuring BP on at least two occasions to define hypertension had significantly lower hypertension prevalence than studies categorizing hypertension based on measurements on a single occasion (4·8% [95%CI 3·3-6·5] vs 8·0% [95% CI 5·9-10·5], p=0·018). Only two studies did not report multiple measures on different occasions or at least 2 measures on one occasion, [34,44] one of which statistically adjusted for this according to a reference standard [44]. When omitting the remaining study, the pooled hypertension prevalence was 7.0 (4.9-9.4) (Supplementary figure 13). Hypertension prevalence was higher in studies using the undefined or other diagnostic standards for the classification of hypertension (9·8% [95%CI 2·0-20·1]) than those using the AAP 2004 or 2017 guidelines (7·2% [95%CI 4·9-9·9], 3·2% [95%CI 2·3-4·4], p=0·003). Reflecting these other methodological considerations, studies assigned a moderate risk of bias score had a higher prevalence of hypertension than those with a lower risk of bias score (11·5% [95%CI 6·3-18·0] vs 5·3% [95%CI 3·9-6·9], p=0·021).

Heterogeneity remained high within each subgroup analysis (>90%). An indication of publication bias by Funnel plot and Egger test statistic was found for the Western Africa region, age, sex, BMI category, and manual BP auscultation.

In univariate meta-regression analysis, no significant associations were found between hypertension prevalence and individual variables, including GDP per capita, which was log-transformed for normality, mean BMI, and mean age (supplementary table 2). In multivariable meta-regression, a significant association with hypertension prevalence was found for country log-GDP (adjusted coefficient: 0·082, 95%CI: 0·014-0·151, p=0·024) and mean age (adjusted coefficient: -0·026, 95%CI: -0·044- -0·008, p=0·010), when also adjusting for mean study BMI (model 2, table 3), with this model accounting for 69·5% of between-study variance (adjusted R^2^). However, these associations were no longer significant when additionally adjusting for methodological characteristics (automated vs manual BP measurement and number of measurement occasions) (model 3, table 3).

**Table 3:** Multivariable meta-regression analyses results for hypertension prevalence

**Model 1**	Coefficient	Standard error	p-value	95% Confidence interval	I^2^ residual (%)		AdjustedR^2^ (%)	
Log-GDP	0·061	0·041	0·164	-0·029-0·150	77·3		16·5	
Mean BMI	-0·005	0·011	0·682	-0·028-0·019	N=15			
**Model 2**								
Log-GDP	0·082	0·031	0·024*	0·014-0·151	60·9			69·5
Mean BMI	0·020	0·012	0·133	-0·007-0·048	N=14			
Mean Age	-0·026	0·008	0·010*	-0·044- -0·008				
**Model 3**								
Log-GDP	0·059	0·050	0·297	-0·707-0·188	79·6	8·2		
Mean BMI	0·002	0·028	0·947	-0·071-0·075	N=11			
Mean Age	-0·011	0·023	0·655	-0·069-0·048				
Nr of measures	-0·016	0·083	0·856	-0·230-0·198				
Auto vs Manual	-0·038	0·085	0·670	-0·257-0·180				

Log-GDP: log-transformed gross domestic product of country; BMI: body mass index; Auto vs Manual: automatic blood pressure measurement (oscillometric) vs manual blood pressure measurement (auscultation).

## 6 Discussion

We found an overall prevalence of hypertension, elevated blood pressure (BP) and combined elevated BP and hypertension in African children and adolescents of 7·5%, 11·4% and 21·7%, respectively. Our analysis showed that hypertension was four times more prevalent in participants classified as overweight or with obesity than in those classified as normal weight.

As our methods were similar to the previous review [10], when comparing the reviews, we were able to find three important insights: (1) The prevalence of hypertension has increased from 5·5% to 7·5%; (2) The prevalence of elevated BP has remained unchanged (12·7% to 11·4%); and (3) The number of studies reporting data on paediatric elevated BP has increased significantly (from 51 papers in 21 years, representing 13 countries, to 53 papers in four years, representing ten countries). As in the previous review, we found significantly higher levels of hypertension in children and adolescents classified as with obesity or overweight. While our subgroup meta-analysis indicated that the prevalence of hypertension in participants that were with obesity/overweight was four times as high as participants with normal BMI, the previous review identified a six-fold increase when comparing these groups [10]. A lack of a significant association between BMI and hypertension prevalence in the meta-regression could be due to the small number of papers included (n=15 to 11), and differences in BMI trends across included studies in terms of age group, sex, and geographical location that we could not entirely correct for. Both reviews are in line with numerous studies showing a connection between overweight and obesity and hypertension and CVD both in Africa and globally [76]. This apparent change in the risk may reflect a change in prevalence of elevated BP, of obesity or of both. Obesity levels amongst children and adolescents in Africa are rapidly growing [7], with North Africa among the regions with the largest absolute increase in the number of children and adolescents with obesity. [7]

This obesity increase in North Africa was mirrored by increased hypertension prevalence. There were significant differences seen between the different African regions (Northern (15·2%), Eastern (9·5%), Central (1·6%), Southern (7·9%) and Western (6·0%)). Though this may result from limited data in this area, with only three studies from Northern Africa included in the meta-analyses, and one in particular (El-Koofy et al [34]) reporting significantly higher levels of hypertension and obesity compared to other studies in the North African region.

We also found no differences in hypertension prevalence between boys and girls. This is different to findings from other world regions, for example, in North America boys were more likely to have high BP compared to girls [19]. This could be driven by underlying cultural, behavioural, and biological factors that are not necessarily the same worldwide. The wide variety of countries, regions, and ages included in this review may have prevented us from identifying population-specific sex differences in hypertension prevalence.

In our multivariable meta-regression analysis, higher country GDP was found to be significantly associated with higher hypertension prevalence, when also adjusting for BMI. Variations in cardiovascular risk factors across different socioeconomic status groups tend to differ in high-income countries vs LMIC, which we are unable to further evaluate in more detail within this pooled meta-analysis [77]. However, the association between GDP and hypertension was lost when additional variables concerning measurement method were added to the model. With this said, countries with a higher GDP may be better equipped both to follow measurement protocols resulting in a lower prevalence and to roll out effective prevention, diagnosis, and treatment programs to ultimately reduce the burden of hypertension. Our results may indicate suppression of the association between log-GDP and hypertension prevalence when not adjusting for age and BMI, due to confounding or selection of studies with available data.

We found no difference in hypertension prevalence between rural and urban areas – as opposed to the previous review, which found prevalence to be higher in rural areas. This may be because an increase in prevalence of risk factors for high blood pressure have become more pervasive in all areas, or it may be that the mix of countries in each study were at different stages of the nutritional and demographic transition. Indeed, studies in adults in the region suggest that urban/rural differences may be country specific. For example, a study in Zambia found rural adults had twice the hypertension prevalence (47%) of urban Zambians (23%) [78], while in Sierra Leone, prevalence in rural or urban areas was similar [79], and in Kenyan women, those in urban areas had higher hypertension prevalence than their rural counterparts [80].

In this systematic review we also investigated the impact of measurement methods on the reported prevalence. The impact of variations in cuff size, measurement number, technique and type of device used are well described and this was evident in our review [81]. Significant differences were seen with type of BP measurement device used (automated oscillometric vs manual auscultation), the number of measurements taken (single vs multiple) and the classification standard used (2004 AAP Fourth Report vs 2017 AAP Clinical Practice vs Other/unclear). This was found to partially account for the high heterogeneity seen between studies, although significant heterogeneity remained in subgroup analyses. These findings highlight several problems in the evaluation of paediatric hypertension in Africa, including the lack of standardized clinical guidelines for the region. Therefore, defining acceptable methods appropriate to African settings is essential for determining the prevalence of hypertension amongst children in this region, which clearly varies widely with the method used.

Of the studies reporting method of BP collection, 21 made use of automated machines while 11 collected BP manually. We found a higher prevalence of hypertension in children and adolescents measured with automated oscillometric devices (oscillometric 8·2%; auscultatory 4·6%), which is in contrast to existing studies in adults [82]. However, a systematic review comparing automated oscillometric and manual auscultatory methods in children did conclude automated oscillometric devices may be suitable for initial screening [83]. This is in line with the most recent (2017) paediatric hypertension guidelines [19]. The use of manual BP devices requires a skilled observer and more time, which may also present a challenge in low recourse settings. Manual auscultatory devices also eliminate the possibility of effective home monitoring. When collecting BP using automated oscillometric devices, it is important to ensure the device is validated for use in children, a factor rarely reported. An additional barrier to both oscillometric and auscultatory methods in children and adolescents is the importance of using paediatric cuffs. Paediatric cuffs are expensive and may not be available in low-resource settings. Similar to the used of validated devices, cuff size is often not reported.

When evaluating BP, the number of measures and measurement occasions is also of importance. This review found that the prevalence of hypertension in studies measuring BP on a single occasion was almost double that of the studies that measured BP on multiple (at least 2) occasions (single 8·0%; multiple 4·8%). While epidemiological studies frequently assess blood pressure utilising 2-3 readings on a single occasion, the 2004 AAP Fourth Report [24] only recommends clinical diagnosis of hypertension if BP is consistently high on three occasions, and the updated 2017 AAP Clinical Practice Guideline [19] recommends clinical diagnostic evaluation and treatment initiation upon high BP measured on multiple occasions. However, settings in which healthcare has limited resources and is less accessible may struggle to implement such recommendations in clinical settings, and our results indicate that this may have implications for the identification of hypertension and, therefore, the appropriate use of resources.

In addition to these methodological differences in the collection of blood pressure, we found significant differences in hypertension prevalence based on the standards used to classify hypertension. Of the 41 studies included in this review, 32 used the 2004 AAP Fourth Report [24] (hypertension prevalence 7·2%), two used the more recent 2017 AAP Clinical Practice [19] (hypertension prevalence 3·2%), and 7 studies either made use of other guidelines or did not clearly report which guidelines were used (hypertension prevalence 9·8%) [84], [85], [86]. The lower prevalence of hypertension in the studies using the 2017 AAP guidelines is unexpected, since they have lower cut-offs. This is likely due to differences in other population characteristics, such as the lower overweight/obesity prevalence, in those two studies. Currently there are no African specific guidelines for the classification of hypertension in children and adolescent. As a result, clinicians and researchers make use of different international guidelines, leading to inconsistent classification of hypertension. While the two most prevalent classification standards used in this review are internationally accepted, their suitability for an African child and adolescent populations remains unknown. A study comparing the 2017 AAP guidelines to that of the 2004 4^th^ report, in 47200 paediatric subjects from an international cohort (China, India, Iran, Korea, Poland, and Tunisia), found that making use of the 2017 AAP guidelines resulted in a 6·3% reduction in elevated BP [87]. However, the prevalence of both stage one (7·9 % increase) and stage two (1·3% increase) hypertension increased [87]. Additionally, a case control sub-study of 1606 subjects from the above mentioned cohort showed that, compared to normotensive children, those reclassified upwards were more likely to have a higher fasting blood glucose and advance lipid profile [87]. This highlights the potential clinical significance of appropriately classifying paediatric hypertension.

Taking into consideration the numerous barriers to effective diagnosis and management of hypertension in African children and adolescents, primary prevention is essential. Given the clear association between obesity and hypertension, programs such as those focusing on weight control via the encouragement of balanced child and maternal nutrition, feeding schemes and promotion of regular exercise may help reduce the development of hypertension. Programs promoting regular and effective home screening may additionally prove a valuable strategy. Furthermore, the education of primary healthcare providers on the importance of regular and accurate BP screening even in resource-constrained environments is required. With this said, the previous review by Noubiap et al [10] similarly suggested the need for primary prevention through population interventions such as weight control, diet modification and the promotion of physical activity, potentially through the use of existing child and maternal health and school programmes. While some programs focused on physical activity, diet and weight have been implemented in Africa, they remain scarce, are often (temporarily) implemented by individual organisations, and are inaccessible for many. However, country-level programs, such as the South African salt legislation, have proven to be effective in eliciting population level changes [88]. It is clear that the prevalence of hypertension is continuing to rise and the need for effective increased primary prevention programmes, including at country-level remains. Without intervention, the continued increase in paediatric hypertension, often tracking into adulthood, [14,15] will increasingly burden often already strained health care systems.

Our results should be viewed within the context of the strengths and limitations. The overall prevalence estimates should be interpreted considering the significant heterogeneity present amongst studies. While potentially due in part to methodological and participant differences between studies, this could not be fully accounted for by subgroup or regression analyses. Furthermore, studies differed in the classification used to define hypertension. As such it is difficult to determine the actual prevalence of hypertension. Moreover, the Egger test for some of the subgroup comparisons was significant, indicating possible presence of publication bias against smaller studies with different (larger or smaller) prevalence estimates. However, the presence of significant between-study heterogeneity can ‘confound’ the assessment of publication bias, so that funnel plots and quantitative measures such as the Egger test cannot be reliably interpreted as measures of publication bias [25,89]. While this review did take into consideration all papers fitting the inclusion criteria, data pertaining to paediatric hypertension was not equally distributed across all regions of Africa, affecting the comparability of the different regions. Moreover, the identified papers only represented ten African countries, three fewer than the previous review [10]. Such unrepresented geographical differences may have impacted our findings, for example for the Northern region for which only three papers were available, one of which had prevalence of 38.9%, likely driving the high prevalence estimate for the region. This highlights the importance of encouraging paediatric hypertension research across all African countries. As the previous review did not report on classification guidelines used we are unable to compare prevalence based on differing guidelines. Lastly, not all studies included in the meta-analysis had data available for the variable(s) of interest, resulting in a lower number of studies included in the meta-analysis and subgroup analyses than in the systematic review.

In conclusion, despite difficulties in determining the true prevalence of hypertension, this review shows a rapidly expanding interest in paediatric hypertension and elevated BP across the continent, suggesting that countries are recognising this growing problem. Further, the review suggests prevalence of hypertension among children and adolescents in Africa has continued to increase over the last three years, in line with increases in obesity in Africa. On a continent plagued by low resources and sub-optimal access to healthcare, prevention and strategies for effective early detection are of paramount importance. Additionally, pan-African guidelines and standards are urgently needed to help guide the measurement, screening, and management of hypertension for children in the region.

## Contributors

SHC and LJW conceived the study and, together with LMS and AKR designed the protocol. SHC conducted the literature search. SHC, LMS and AKR selected the studies and extracted the relevant data. LMS, AKR and IM analysed the data. SHC, LMS and AKR wrote the original draft and SHC, LMS, AKR, SN, JD, SAN, LJW critically revised and edited successive drafts of the paper. All authors gave final approval of the version to be submitted.

## Data Sharing

All data shall be made available upon reasonable request to the corresponding author and all articles included in the analysis are available online.

## Funding

This research was funded in part by the Wellcome Trust [Grant number:214082/Z/18/Z]. LJW and SAN are supported by the DSI-NRF Centre of Human Development at the University of the Witwatersrand.

## Declaration of Competing Interest

We declare no competing interests.

## Appendix Supplementary materials

Image, application 1

Image, application 2

## References

[1] Abegunde DO, Mathers CD, Adam T, Ortegon M, Strong K. (2007). The burden and costs of chronic diseases in low-income and middle-income countries. The Lancet.

[2] World Health Organisation. Cardiovascular diseases (CVDs) 2017 [Available from: https://www.who.int/en/news-room/fact-sheets/detail/cardiovascular-diseases-(cvds).

[3] Juma K, Juma PA, Shumba C, Otieno P, Asiki G. (2019). Non-communicable diseases and urbanization in African cities: A narrative review. Public Health in Developing Countries-Challenges and Opportunities.

[4] Pirgon Ö, Aslan N. (2015). The role of urbanization in childhood obesity. Journal of clinical research in pediatric endocrinology.

[5] Pieters M, Vorster HH. (2008). Nutrition and hemostasis: a focus on urbanization in South Africa. *Molecular nutrition & food research*.

[6] Pranata R, Vania R, Tondas AE, Setianto B, Santoso A. (2020). A time-to-event analysis on air pollutants with the risk of cardiovascular disease and mortality: A systematic review and meta-analysis of 84 cohort studies. Journal of Evidence-Based Medicine.

[7] Abarca-Gómez L, Abdeen ZA, Hamid ZA, Abu-Rmeileh NM, Acosta-Cazares B, Acuin C (2017). Worldwide trends in body-mass index, underweight, overweight, and obesity from 1975 to 2016: a pooled analysis of 2416 population-based measurement studies in 128· 9 million children, adolescents, and adults. The Lancet.

[8] Leggio M, Lombardi M, Caldarone E, Severi P, D'emidio S, Armeni M (2017). The relationship between obesity and hypertension: an updated comprehensive overview on vicious twins. Hypertension Research.

[9] Kannel WB. (1996). Blood pressure as a cardiovascular risk factor: prevention and treatment. JAMA.

[10] Noubiap JJ, Essouma M, Bigna JJ, Jingi AM, Aminde LN, Nansseu JR. (2017). Prevalence of elevated blood pressure in children and adolescents in Africa: a systematic review and meta-analysis. The Lancet Public health.

[11] Bromfield S, Muntner P. (2013). High blood pressure: the leading global burden of disease risk factor and the need for worldwide prevention programs. Current hypertension reports.

[12] Chen X, Wang Y. (2008). Tracking of blood pressure from childhood to adulthood: a systematic review and meta-regression analysis. Circulation.

[13] Toschke AM, Kohl L, Mansmann U, Von Kries R. (2010). Meta-analysis of blood pressure tracking from childhood to adulthood and implications for the design of intervention trials. Acta Paediatrica.

[14] Yang L, Magnussen CG, Yang L, Bovet P, Xi B. (2020). Elevated blood pressure in childhood or adolescence and cardiovascular outcomes in adulthood: a systematic review. Hypertension.

[15] Högström G, Nordström A, Eriksson M, Nordström P. (2015). Risk factors assessed in adolescence and the later risk of stroke in men: a 33-year follow-up study. Cerebrovascular Diseases.

[16] Rundle AG, Factor-Litvak P, Suglia SF, Susser ES, Kezios KL, Lovasi GS (2020). Tracking of obesity in childhood into adulthood: effects on body mass index and fat mass index at age 50. Childhood Obesity.

[17] Hoy D, Brooks P, Woolf A, Blyth F, March L, Bain C (2012). Assessing risk of bias in prevalence studies: modification of an existing tool and evidence of interrater agreement. Journal of clinical epidemiology.

[18] Macaulay S, Dunger DB, Norris SA. (2014). Gestational diabetes mellitus in Africa: a systematic review. PloS one.

[19] Flynn JT, Kaelber DC, Baker-Smith CM, Blowey D, Carroll AE, Daniels SR (2017). Clinical practice guideline for screening and management of high blood pressure in children and adolescents. Pediatrics.

[20] United Nations Statistics Division (2021). Africa geosche.

[21] The Word Bank. GDP ranking 2020 [Available from: https://datacatalog.worldbank.org/dataset/gdp-ranking.

[22] Barendregt JJ, Doi SA, Lee YY, Norman RE, Vos T. (2013). Meta-analysis of prevalence. Journal Epidemiol Community Health.

[23] Higgins JP, Thompson SG. (2002). Quantifying heterogeneity in a meta-analysis. Statistics in Medicine.

[24] Falkner B, Daniels SR, Flynn JT, Gidding S, Green LA, Ingelfinger JR (2004). The fourth report on the diagnosis, evaluation, and treatment of high blood pressure in children and adolescents. Pediatrics.

[25] Hayashino Y, Noguchi Y, Fukui T. (2005). Systematic evaluation and comparison of statistical tests for publication bias. Journal of Epidemiology.

[26] Letamo G, Keetile M, Navaneetham K, Phatsimo M. (2017). Prevalence and correlates of self-reported chronic non-communicable diseases in Botswana: a cross-sectional study. International Health.

[27] Bhimma R, Naicker E, Gounden V, Nandlal L, Connolly C, Hariparshad S. (2018). Prevalence of Primary Hypertension and Risk Factors in Grade XII Learners in KwaZulu-Natal, South Africa. International Journal of Hypertension.

[28] Mokgwathi M, Mwita JC. (2020). Prevalence of hypertension and selected cardiovascular risk factors among adolescents in selected rural and urban secondary schools in Botswana. Cardiovascular Journal of Africa.

[29] South AM, Nixon PA, Chappell MC, Diz DI, Russell GB, Shaltout HA (2019). Obesity is Associated with Higher Blood Pressure and Higher Levels of Angiotensin II but Lower Angiotensin-(1-7) in Adolescents Born Preterm. The Journal of Pediatrics.

[30] South AM, Nixon PA, Chappell MC, Diz DI, Russell GB, Jensen ET (2019). Renal function and blood pressure are altered in adolescents born preterm. Pediatric Nephrology.

[31] Muyumba EK, Nkulu DN, Mukeng CK, Musung JM, Kakoma PK, Kakisingi CN (2018). Oscillometric blood pressure by age and height for non overweight children and adolescents in Lubumbashi, Democratic Republic of Congo. BMC Cardiovascular Disorders.

[32] Mphekgwana PM, Monyeki KD, Makgopa HM, Makgae PJ. (2020). Multiple Points Change in the Association of Blood Pressure Subtypes with Anthropometric Indices of Adiposity among Children in a Rural Population. Children.

[33] Musa DI, Toriola AL, Goon DT, SU Jonathan (2020). Association of fitness and fatness with clustered cardiovascular disease risk factors in Nigerian adolescents. International Journal of Environmental Research and Public Health.

[34] El-Koofy N, Mehawed H, Elbarbary M-A, Garhy ASE, Shaba M, Fouad H. (2020). Use of Anthropometry Versus Ultrasound for the Assessment of Body Fat and Comorbidities in Children With Obesity. Journal of pediatric gastroenterology and nutrition.

[35] Elseifi OS, Abdelrahman DM, Mortada EM. (2020). Effect of a nutritional education intervention on breakfast consumption among preparatory school students in Egypt. I*nternational Journal of Public Health*.

[36] Hassana NE, El Shebinib SM, El-Masrya SA, Ahmedb NH, Alia MM, El-Saeedc GSM (2019). Association between dietary sodium, calcium, saturated fat and blood pressure in obese Egyptian adolescents. Egyptian Pediatric Association Gazette.

[37] Hassan NE, El Ashmawi AA, El-Masry SA, Zarouk WA, Mira MF, El-Saeed GSM (2019). Metabolic syndrome in a sample of Egyptian adolescent girls and its association with apolipoprotein E. Journal of Paediatrics & Child Health.

[38] Sherif EM, El Maksood AAA, Youssef OI, Salah El-Din NY, Khater OKM (2019). Soluble urokinase plasminogen activator receptor in type 1 diabetic children, relation to vascular complications. Journal of Diabetes and Its Complications.

[39] Katamba G, Agaba DC, Migisha R, Namaganda A, Namayanja R, Turyakira E. (2020). Prevalence of hypertension in relation to anthropometric indices among secondary adolescents in Mbarara, Southwestern Uganda. Italian Journal of Pediatrics.

[40] Leyvraz M, Wahlen R, Bloetzer C, Paradis G, Bovet P, Chiolero A (2018). Persistence of elevated blood pressure during childhood and adolescence: a school-based multiple cohorts study. Journal of Hypertension.

[41] Muhihi AJ, Njelekela MA, Chillo O, Lujani B, Maghembe M, Ngarashi D (2018). Elevated blood pressure among primary school children in Dar es salaam, Tanzania: Prevalence and risk factors. BMC Pediatrics.

[42] Nakiriba R, Mayega RW, Piloya T, Nabukeera-Barungi N, Idro R. (2018). Prevalence and factors associated with dysglycemia among girls in selected boarding secondary schools in Wakiso District, Uganda. *Adolescent Health*. Medicine and Therapeutics.

[43] Nyangasa MA, Buck C, Kelm S, Sheikh MA, Brackmann KL, Hebestreit A. (2019). Association between cardiometabolic risk factors and body mass index, waist circumferences and body fat in a Zanzibari cross-sectional study. BMJ open.

[44] Gerber M, Müller I, Walter C, du Randt R, Adams L, Gall S (2018). Physical activity and dual disease burden among South African primary schoolchildren from disadvantaged neighbourhoods. Preventive Medicine.

[45] Sekokotla MA, Goswami N, Sewani-Rusike CR, Iputo JE, Nkeh-Chungag BN (2017). Prevalence of metabolic syndrome in adolescents living in Mthatha, South Africa. Therapeutics and Clinical Risk Management.

[46] Matjuda EN, Engwa GA, Letswalo PB, Mungamba MM, Sewani-Rusike CR, BN Nkeh-Chungag (2020). Association of Hypertension and Obesity with Risk Factors of Cardiovascular Diseases in Children Aged 6-9 Years Old in the Eastern Cape Province of South Africa. Children.

[47] Chungag A, Tata CM, Sewani-Rusike CR, Nel W, Nkeh-Chungag BN (2019). Ellisras Longitudinal Study 2017: association of hypertension with increasing levels of adiposity in 10- to 14-year-old boys and girls in the Eastern Cape (ELS 31). Cardiovascular Journal of Africa.

[48] Sebati B, Monyeki K, Makgae P. (2020). An Assessment of the Relationship between Anthropometric Parameters and Blood Pressure among Polokwane Private School Children. Children.

[49] Nkwana MR, Monyeki KD, Monyeki SM, Makata TT, Monyeki JM. (2019). Ellisras Longitudinal Study 2017: the association of fat patterning with blood pressure in Polokwane private school children aged five to 15 years (ELS 22). Cardiovascular Journal of Africa.

[50] Masocha V, Monyeki MA, Czyż SH. (2020). Longitudinal relationships between changes in body composition and changes in selected metabolic risk factors (abdominal obesity and blood pressure) among South African adolescents. PeerJ.

[51] Raphadu TT, Van Staden M, Dibakwane WM, Monyeki KD. (2020). A Non-Invasive Investigation into the Prevalence of Higher than Normal Blood Pressure, Hypertension and the Association between Blood Pressure and Body Weight in Male and Female Adolescents in the Polokwane Local Municipality, Limpopo-South Africa: A Cross-Sectional Study. Children.

[52] Negash S, Agyemang C, Matsha TE, Peer N, Erasmus RT, Kengne AP. (2017). Differential prevalence and associations of overweight and obesity by gender and population group among school learners in South Africa: a cross-sectional study. BMC Obesity.

[53] Azupogo F, Osendarp SJM, Feskens EJM, Brouwer ID, Abizari AR, Aurino E (2020). Malnutrition, hypertension risk, and correlates: An analysis of the 2014 ghana demographic and health survey data for 15–19 years adolescent boys and girls. Nutrients.

[54] Amponsem-Boateng C, Zhang W, Oppong TB, Opolot G, Kumi Duodu Kyere E (2019). A cross-sectional study of risk factors and hypertension among adolescent Senior High School students. Diabetes, metabolic syndrome and obesity: targets and therapy.

[55] Alicke M, Boakye-Appiah JK, Abdul-Jalil I, Henze A, van der Giet M, Schulze MB (2017). Adolescent health in rural Ghana: A cross-sectional study on the co-occurrence of infectious diseases, malnutrition and cardio-metabolic risk factors. PLoS ONE.

[56] Ibrahim OR, Afolabi JK, Adedoyin OT, Ojuawo AI. (2019). Prevalence and risk factors for hypertension among school children in Ilorin, Northcentral Nigeria. Journal of Family and Community Medicine.

[57] Abu O, Raji Y, Amodu O. (2019). Risk factors for chronic kidney disease among in-school adolescents in Ibadan, Southwest, Nigeria. Sahel Medical Journal.

[58] Abiodun O, Ladele A, Olu-Abiodun O, Ashipa T. (2019). Hypertension among adolescents in Nigeria: a retrospective study of adolescent university freshmen. International Journal of Adolescent Medicine and Health.

[59] Emmanuel EE, Dada SA, Amu EO, Aduayi VA, Atoyebi OA, Marcus O (2017). Hypertension and its correlates among in-school adolescents in Ekiti State, South-west, Nigeria. Asian Journal of Medical Sciences.

[60] Ezeudu CE, Chukwuka JO, Ebenebe JC, Igwe WC, Egbuonu I. (2018). Hypertension and prehypertension among adolescents attending secondary schools in urban area of South-East, Nigeria. Pan African Medical Journal.

[61] Amadi OF, Okeke IB, Ndu IK, Ekwochi U, Nduagubam OC, Ezenwosu OU (2019). Hypertension in Children: Could the Prevalence be on the Increase?. Nigerian medical journal: Journal of the Nigeria Medical Association.

[62] Omisore AG, Omisore B, Abioye-Kuteyi EA, Bello IS, Olowookere SA. (2018). In-school adolescents' weight status and blood pressure profile in South-western Nigeria: Urban-rural comparison. BMC Obesity.

[63] Adeomi A, Adelusi I, Adedeji P, Awofeso A, Oroleye O, Gbadegesin D. (2019). Nutritional status and Cardiometabolic health among adolescents; findings from southwestern Nigeria. BMC Nutrition.

[64] Ukoh U, Ujunwa F, Muoneke U, Manyike P, Okike C, Ibe B. (2020). Oscillometric blood pressure profile of adolescent secondary school students in Abakaliki metropolis. Annals of African Medicine.

[65] Isezuo KO, Jiya NM, Ibitoye PK, Sani UM, Yusuf T, Garba BI (2018). Blood pressure pattern and the relationship with body mass index among apparently healthy secondary - School students in Sokoto Metropolis, Nigeria. SAJCH South African Journal of Child Health.

[66] Yilgwan C, Hyacinth H, Ige O, Abok I, Yilgwan G, John C (2017). Cardiovascular disease risk profile in Nigerian school children. Sahel Medical Journal.

[67] Sadoh WE, Israel-Aina YT, Sadoh AE, Uduebor JE, Shaibu M, Ogonor E (2017). Comparison of obesity, overweight and elevated blood pressure in children attending public and private primary schools in Benin City, Nigeria. Nigerian Journal of Clinical Practice.

[68] Chedjou-Nono E, Sap S, Choukem S-P, Tetanye IN, Nebongo D, Ndombo OK (2017). Cardiometabolic profile of obese children in a sub-Saharan African setting: a cross-sectional study. BMC Pediatrics.

[69] Chelo D, Mah EM, Chiabi EN, Chiabi A, Koki Ndombo PO, Kingue S (2019). Prevalence and factors associated with hypertension in primary school children, in the centre region of Cameroon. Translational Pediatrics.

[70] Benmohammed K, Valensi P, Nguyen MT, Benmohammed F, Benlatreche M, Benembarek K (2020). Influence of waist circumference on blood pressure status in non-obese adolescents. International J*ournal of Adolescent Medicine & Health*.

[71] Wariri O, Jalo I, Bode-Thomas F. (2018). Discriminative ability of adiposity measures for elevated blood pressure among adolescents in a resource-constrained setting in northeast Nigeria: a cross-sectional analysis. BMC Obesity.

[72] Nsanya MK, Kavishe BB, Katende D, Mosha N, Hansen C, Nsubuga RN (2019). Prevalence of high blood pressure and associated factors among adolescents and young people in Tanzania and Uganda. Journal of Clinical Hypertension.

[73] Nqweniso S, Walter C, du Randt R, Aerts A, Adams L, Degen J (2020). Prevention of Overweight and Hypertension through Cardiorespiratory Fitness and Extracurricular Sport Participation among South African Schoolchildren. Sustainability.

[74] Schoenbuchner SM, Moore SE, Johnson W, Ngum M, Sonko B, Prentice A (2018). In rural Gambia, do adolescents have increased nutritional vulnerability compared with adults?. Annals of the New York Academy of Sciences.

[75] Gomwe H, Seekoe E, Lyoka P, Marange CS. (2019). The relationship between body composition and blood pressure among primary school children in Eastern Cape province, South Africa. African Journal of Primary Health Care & Family Medicine.

[76] Lee M-R, Lim Y-H, Hong Y-C. (2018). Causal association of body mass index with hypertension using a Mendelian randomization design. Medicine.

[77] Rosengren A, Subramanian S, Islam S, Chow CK, Avezum A, Kazmi K (2009). Education and risk for acute myocardial infarction in 52 high, middle and low-income countries: INTERHEART case-control study. Heart.

[78] Rush KL, Goma FM, Barker JA, Ollivier RA, Ferrier MS, Singini D. (2018). Hypertension prevalence and risk factors in rural and urban Zambian adults in western province: a cross-sectional study. The Pan African Medical Journal.

[79] Odland ML, Bockarie T, Wurie H, Ansumana R, Lamin J, Nugent R (2020). Prevalence and access to care for cardiovascular risk factors in older people in Sierra Leone: a cross-sectional survey. BMJ open.

[80] Chowdhury MAB, Epnere K, Haque MA, Mkuu RS. (2020). Urban rural differences in prevalence and risk factors of self-reported hypertension among Kenyan women: a population-based study. Journal of Human Hypertension.

[81] Cloutier L, Schiffrin EL. (2012). Hypertension prevalence and control: impact of method of blood pressure measurement. *Current Cardiovascular Risk Report*s.

[82] Landgraf J, Wishner SH, Kloner RA. (2010). Comparison of automated oscillometric versus auscultatory blood pressure measurement. The American Journal of Cardiology.

[83] Duncombe SL, Voss C, Harris KC. (2017). Oscillometric and auscultatory blood pressure measurement methods in children: a systematic review and meta-analysis. Journal of Hypertension.

[84] Neuhauser HK, Thamm M, Ellert U, Hense HW, Rosario AS. (2011). Blood pressure percentiles by age and height from nonoverweight children and adolescents in Germany. Pediatrics.

[85] Chobanian AV, Bakris GL, Black HR, Cushman WC, Green LA, Izzo Jr JL (2003). Seventh report of the joint national committee on prevention, detection, evaluation, and treatment of high blood pressure. Hypertension.

[86] Jolliffe CJ, Janssen I. (2007). Development of age-specific adolescent metabolic syndrome criteria that are linked to the Adult Treatment Panel III and International Diabetes Federation criteria. Journal of the American College of Cardiology.

[87] Yang L, Kelishadi R, Hong YM, Khadilkar A, Nawarycz T, Krzywińska-Wiewiorowska M (2019). Impact of the 2017 American Academy of Pediatrics guideline on hypertension prevalence compared with the Fourth Report in an international cohort. Hypertension.

[88] Charlton K, Corso B, Ware L, Schutte AE, Wepener L, Minicuci N (2021). Effect of South Africa's interim mandatory salt reduction programme on urinary sodium excretion and blood pressure. Preventive Medicine Reports.

[89] Peters JL, Sutton AJ, Jones DR, Abrams KR, Rushton L, Moreno SG. (2010). Assessing publication bias in meta-analyses in the presence of between-study heterogeneity. Journal of the Royal Statistical Society: Series A (Statistics in Society).

[90] Bouhenni H, Daoudi H, Djemai H, Noirez P, Rouabah A, Vitiello D (2017). Relationships between metabolic profile, hypertension and uric acid with cardiometabolic risk in adolescents with abdominal obesity: impact of geodemographic factors on the prevalence of abdominal obesity. International journal of adolescent medicine and health.

[91] Lule SA, Namara B, Akurut H, Muhangi L, Lubyayi L, Nampijja M (2019). Are birthweight and postnatal weight gain in childhood associated with blood pressure in early adolescence? Results from a Ugandan birth cohort. International Journal of Epidemiology.

[92] Matjuda EN, Sewani-Rusike CR, Anye SNC, Engwa GA, Nkeh-Chungag BN (2020). Relationship between High Blood Pressure and Microalbuminuria in Children Aged 6-9 Years in a South African Population. Children.

[93] Sekgala M, Monyeki K, Mogale M, Ramoshaba N. (2017). Performance of blood pressure to height ratio as a screening tool for elevated blood pressure in rural children: Ellisras Longitudinal Study. Journal of Human Hypertension.

[94] Mokwatsi GG, Schutte AE, Kruger R. (2017). Ethnic differences regarding arterial stiffness of 6-8-year-old black and white boys. Journal of Hypertension.

[95] Houle B, Rochat TJ, Newell M-L, Stein A, Bland RM. (2019). Breastfeeding, HIV exposure, childhood obesity, and prehypertension: A South African cohort study. PLoS Medicine.

[96] Jobe M, Agbla SC, Prentice AM, Hennig BJ. (2017). High blood pressure and associated risk factors as indicator of preclinical hypertension in rural West Africa: A focus on children and adolescents in The Gambia. Medicine.

[97] Okpokowuruk FS, Akpan MU, Ikpeme EE. (2017). Prevalence of hypertension and prehypertension among children and adolescents in a semi-urban area of Uyo Metropolis, Nigeria. Pan African Medical Journal.

[98] Akinbodewa AA, Adejumo AO, Lamidi OA, Adeyemi O. (2020). Clustering of Cardiometabolic Risk Factors among Children and Adolescents in a Rural Community in Ondo, Southwest Nigeria. Journal of Tropical Pediatrics.

