# Supplementary material for: Paediatric Hypertension in Africa: A Systematic Review and Meta-Analysis
Source: eClinicalMedicine. 2021 Dec 6;43:101229. doi: 10.1016/j.eclinm.2021.101229 (PMC8665406; doi:10.1016/j.eclinm.2021.101229)
Supplement: Supplementary file 1 [file mmc1.docx]

Ms. Ref. No.:  eclinm-D-21-00890R1
Title: Paediatric Hypertension in Africa: A Systematic Review and Meta-Analysis

Supplementary File 1: Search strategy

Supplementary Table 1: Predefined data extraction form

Supplementary Table 2: Univariate regression analysis results for hypertension prevalence

Supplementary Table 3. Risk of Bias results

Supplementary Figure 1: Funnel plots for meta-analysis for prevalence of a) hypertension b) elevated blood pressure and c) combined elevated blood pressure and hypertension.

Supplementary Figure 2: Funnel plots for hypertension meta-analysis by subgroup: Africa region

Supplementary Figure 3: Funnel plots for hypertension meta-analysis by subgroup: Geographical setting

Supplementary Figure 4: Funnel plots for hypertension meta-analysis by subgroup: Timing of data collection

Supplementary Figure 5: Funnel plots for hypertension meta-analysis by subgroup: Age group

Supplementary Figure 6: Funnel plots for hypertension meta-analysis by subgroup: Sex

Supplementary Figure 7: Funnel plots for hypertension meta-analysis by subgroup: BMI category

Supplementary Figure 8: Funnel plots for hypertension meta-analysis by subgroup: BP methodology

Supplementary Figure 9: Funnel plots for hypertension meta-analysis by subgroup: Number of measurement occasions

Supplementary Figure 10: Funnel plots for hypertension meta-analysis by subgroup: Standards used for classification of HTN

Supplementary Figure 11: Funnel plots for hypertension meta-analysis by subgroup: Sample size

Supplementary Figure 12: Funnel plots for hypertension meta-analysis by subgroup: Risk of bias score

Supplementary Figure 13: Meta-analysis for hypertension prevalence excluding study (ref: 44) not reporting, or adjusting for lack of, multiple measures
