# Supplementary material for: Paediatric Hypertension in Africa: A Systematic Review and Meta-Analysis
Source: eClinicalMedicine. 2021 Dec 6;43:101229. doi: 10.1016/j.eclinm.2021.101229 (PMC8665406; doi:10.1016/j.eclinm.2021.101229)
Supplement: Supplementary file 2 [file mmc2.docx]

**Supplementary file 1:**

**Search Strategy:**

(hypertension or blood pressure or systolic hypertension or diastolic hypertension or systolic blood pressure or diastolic blood pressure) AND

(child or children or childhood or adolescent or teens or teenage or youth or infant or paediatric or pediatric) AND

(african or africa or north africa or northern africa or central africa or middle africa or southern africa or east african or eastern african or west africa or western africa or algeria or angola or portuguese west africa or benin or dahomey or botswana or bechuanaland burkina faso or upper volta or haute voltaire or burundi or cameroon or cabo verde or cape verde central african republic or chad or comoros or congo or moyen congo or french congo or democratic republic of congo or zaire or cote d’ivoire or ivory coast or djibouti or french territory of afar issas or equatorial guinea or rio muni or fernando po or egypt or eritrea or ethiopia or gabon or gambia or ghana or guinea or french guinea or guinea bissau or portuguese guinea or kenya or lesotho or basutoland or liberia or libya or madagascar or malawi or nyasaland or mali or mauritania or mauritius or morocco or mozambique or portuguese east africa or namibia or south west africa or niger or nigeria or rwanda or Ruanda urundi or saharawi arab democratic republic or sao tome principe or senegal or seychelles or sierra leone or somalia or british somaliland or south africa or south sudan or sudan or kingdom of swaziland or eswatini or tanzania or tanganyika or togo or tunisia or uganda or zambia or northern rhodesia or zimbabwe or rhodesia or western sahara) NOT (african american or black americans)

**Supplementary table 1. Predefined data extraction form**

| Author | Year published | Year Collected | Country | Region | Geographical Setting | GDP per capita at collection | Sample size | Age range | Mean Age |
| --- | --- | --- | --- | --- | --- | --- | --- | --- | --- |
| - | - | - | - | - | - | - | - | - | - |
| Males | Females | Prevalence HTN (%) | Prevalence HTN (n) | Prevalence Elevated BP (%) | Prevalence Elevated BP (n) | Prevalence combined HTN and elevated BP (%) | Prevalence combined HTN and elevated BP (n) | % Normal BMI | % Overweight |
| - | - | - | - | - | - | - | - | - | - |
| % Obese | % Overweight + Obese | HTN per weight category | HTN per sex category | Relevant Associations | HTN classification used | Automated vs manual measurement | Number of occasions measurement | Risk of bias score | Additional Notes |
| - | - | - | - | - | - | - | - | - | - |

**Supplementary table 2. Univariate regression analysis results for hypertension prevalence**

| **Variable** | **Coefficient** | **Standard error** | **p-value** | **95% Confidence interval** | **I^2^ residual (%)** | **Adjusted R^2^ (%)** |
| --- | --- | --- | --- | --- | --- | --- |
| Log-GDP (n=35) | 0·016 | 0·023 | 0·496 | -0·031-0·063 | 91·3 | -3·15 |
| Mean BMI (n=19) | -0·005 | 0·009 | 0·598 | -0·024-0·014 | 78·1 | -0·69 |
| Mean age (n=29) | -0·002 | 0·005 | 0·735 | -0·012-0·009 | 90·0 | -5·3 |
| Urb/rural (n=20)  1=urban, 2= rural | -0·051 | 0·031 | 0·118 | -0·117-0·014 | 75·0 | 12·1 |
| Auto/Manual  (n=32)  1=auto, 2=manual | -0·041 | 0·024 | 0·102 | -0·091-0·009 | 69·6 | 7·5 |
| Nr of measures (n=36)  1=1; 2=multiple | -0·035 | 0·025 | 0·117 | -0·5086-0·017 | 71·8 | 1·7 |
| Standards (n=38)  1=4^th^ report, 2=other | 0·024 | 0·032 | 0·449 | -0·040-0·088 | 89·4 | -0·9 |

**Supplementary table 3. Risk of Bias**

| **Author** | **Criteria 1** | **Criteria 2** | **Criteria 3** | **Criteria 4** | **Criteria 5** | **Criteria 6** | **Criteria 7** | **Criteria 8** | **Criteria 9** | **Criteria 10** | **Total** | **Classification** |
| --- | --- | --- | --- | --- | --- | --- | --- | --- | --- | --- | --- | --- |
| Bouhenni et al[^90^](#_ENREF_90) | High | High | Low | Low | Low | Low | Low | Low | Low | Low | 8 | Low |
| Gomwe et al[^75^](#_ENREF_75) | High | Low | Low | Low | Low | Low | Low | Low | Low | Low | 9 | Low |
| Sherif et al[^38^](#_ENREF_38) | High | High | High | Low | Low | Low | High | Low | Low | Low | 6 | Moderate |
| Abu et al[^57^](#_ENREF_57) | High | High | Low | Low | Low | Low | Low | Low | Low | Low | 8 | Low |
| Matjuda et al[^92^](#_ENREF_92) | High | High | High | Low | Low | Low | Low | Low | Low | High | 6 | Moderate |
| Nqweniso et al[^73^](#_ENREF_73) | High | High | High | Low | Low | Low | Low | Low | Low | Low | 7 | Moderate |
| Sekokotla et al[^45^](#_ENREF_45) | High | High | High | Low | Low | Low | Low | Low | Low | Low | 7 | Moderate |
| Katamba et al[^39^](#_ENREF_39) | High | Low | High | Low | Low | Low | Low | Low | Low | Low | 8 | Low |
| Okpokowuruk et al[^97^](#_ENREF_97) | High | High | Low | Low | Low | Low | Low | Low | Low | Low | 8 | Low |
| Nsanya et al[^72^](#_ENREF_72) | High | Low | High | High | Low | Low | Low | Low | Low | Low | 7 | Moderate |
| Ibrahim et al[^56^](#_ENREF_56) | High | Low | High | Low | Low | Low | Low | Low | Low | Low | 8 | Low |
| Chelo et al[^69^](#_ENREF_69) | High | Low | Low | High | Low | Low | Low | Low | Low | Low | 8 | Low |
| Nakiriba et al[^42^](#_ENREF_42) | High | Low | Low | Low | Low | Low | Low | Low | Low | High | 8 | Low |
| Gerber et al[^44^](#_ENREF_44) | High | High | High | Low | Low | Low | Low | Low | Low | Low | 7 | Moderate |
| Leyvraz et al[^40^](#_ENREF_40) | Low | High | High | Low | Low | Low | Low | Low | Low | Low | 8 | Low |
| Sekgala et al[^93^](#_ENREF_93) | High | Low | Low | Low | Low | Low | Low | Low | Low | High | 8 | Low |
| Schoenbuchner et al[^74^](#_ENREF_74) | High | High | Low | Low | Low | Low | Low | Low | Low | High | 7 | Moderate |
| Nkwana et al[^49^](#_ENREF_49) | High | Low | Low | Low | Low | High | Low | Low | Low | Low | 8 | Low |
| Chungag et al[^47^](#_ENREF_47) | High | High | High | Low | Low | High | Low | Low | Low | Low | 6 | Moderate |
| Mphekgwana et al[^32^](#_ENREF_32) | High | Low | Low | Low | Low | Low | Low | Low | Low | Low | 9 | Low |
| Mokwatsi at al[^94^](#_ENREF_94) | High | High | High | Low | Low | Low | Low | Low | Low | Low | 7 | Moderate |
| Jobe et al[^96^](#_ENREF_96) | High | High | Low | Low | Low | Low | Low | Low | Low | Low | 8 | Low |
| Abiodun et al[^58^](#_ENREF_58) | HIgh | High | High | Low | High | Low | Low | Low | Low | Low | 6 | Moderate |
| Emmanuel et al[^59^](#_ENREF_59) | High | High | Low | Low | Low | Low | Low | Low | Low | Low | 8 | Low |
| Ezeudu et al[^60^](#_ENREF_60) | HIgh | Low | High | Low | Low | Low | Low | Low | Low | Low | 8 | Low |
| Amadi et al[^61^](#_ENREF_61) | High | High | High | Low | Low | Low | Low | Low | Low | Low | 7 | Moderate |
| Benmohammed et al[^70^](#_ENREF_70) | High | High | Low | Low | Low | Low | Low | Low | Low | Low | 8 | Low |
| Omisore et al[^62^](#_ENREF_62) | High | Low | High | Low | Low | Low | Low | Low | Low | Low | 8 | Low |
| Masocha et al[^50^](#_ENREF_50) | High | High | High | Low | Low | High | Low | Low | Low | High | 5 | High |
| Azupogo et al[^53^](#_ENREF_53) | Low | Low | Low | Low | Low | Low | Low | Low | Low | High | 9 | Low |
| Hassan et al[^37^](#_ENREF_37) | High | High | High | Low | Low | Low | Low | Low | Low | Low | 7 | Moderate |
| Adeomi et al [^63^](#_ENREF_63) | High | High | Low | Low | Low | Low | Low | Low | Low | Low | 8 | Low |
| Ukoh et al[^64^](#_ENREF_64) | High | High | Low | Low | Low | Low | Low | Low | Low | Low | 8 | Low |
| El-Koofy et al[^34^](#_ENREF_34) | High | High | High | Low | Low | Low | Low | Low | Low | Low | 7 | Moderate |
| Wariri et al[^71^](#_ENREF_71) | High | Low | Low | Low | Low | Low | Low | Low | Low | High | 8 | Low |
| Negash et al[^52^](#_ENREF_52) | High | Low | Low | Low | Low | Low | Low | Low | Low | Low | 9 | Low |
| Elseifi et al[^35^](#_ENREF_35) | High | High | Low | Low | Low | Low | Low | Low | Low | Low | 8 | Low |
| Muhihi et al[^41^](#_ENREF_41) | High | High | High | Low | Low | Low | Low | Low | Low | Low | 7 | Moderate |
| Matjuda et al[^46^](#_ENREF_46) | High | High | High | Low | Low | Low | Low | Low | Low | Low | 7 | Moderate |
| Amponsem-Boateng et al[^54^](#_ENREF_54) | High | High | High | Low | Low | Low | Low | Low | Low | Low | 7 | Moderate |
| Raphadu et al[^51^](#_ENREF_51) | High | High | High | Low | Low | Low | Low | Low | Low | Low | 7 | Moderate |
| Alicke et al[^55^](#_ENREF_55) | High | High | High | Low | Low | Low | Low | Low | Low | Low | 7 | Moderate |
| Sebati et al[^48^](#_ENREF_48) | High | High | High | Low | Low | Low | Low | Low | Low | Low | 7 | Moderate |
| Lule et al[^91^](#_ENREF_91) | High | High | Low | Low | Low | Low | Low | Low | Low | Low | 8 | Low |
| Nyangasa et al[^43^](#_ENREF_43) | High | Low | Low | Low | Low | Low | Low | Low | Low | Low | 9 | Low |
| Hassana et al[^36^](#_ENREF_36) | High | High | High | Low | Low | Low | Low | Low | Low | Low | 7 | Moderate |
| Musa et al[^33^](#_ENREF_33) | High | High | Low | Low | Low | Low | Low | Low | Low | Low | 7 | Moderate |
| Isezuo et al[^65^](#_ENREF_65) | High | High | High | Low | Low | Low | Low | Low | Low | Low | 7 | Moderate |
| Houle et al[^95^](#_ENREF_95) | High | High | Low | Low | Low | Low | Low | Low | Low | Low | 8 | Low |
| Chedjou-Nono et al[^68^](#_ENREF_68) | High | Low | High | Low | Low | Low | Low | Low | Low | Low | 8 | Low |
| Akinbodewa et al[^98^](#_ENREF_98) | High | High | High | Low | Low | Low | Low | Low | Low | Low | 7 | Moderate |
| Sadoh et al | High | High | High | Low | Low | Low | Low | Low | Low | High | 6 | Moderate |
| Yilgwan et al[^66^](#_ENREF_66) | High | High | Low | Low | Low | Low | Low | Low | Low | Low | 8 | Low |

**Criteria:**

1. Was the study’s target population a close representation of the national population in relation to relevant variables, e.g. age, sex, occupation?
2. Was the sampling frame a true or close representation of the target population?
3. Was some form of random selection used to select the sample, OR, was a census undertaken?
4. Was the likelihood of non-response as minimal?
5. Were data collected directly from the subjects (as opposed to a proxy)?
6. Was an acceptable case definition used in the study?
7. Was the study instrument that measured the parameter of interest (e.g. prevalence of low back pain) shown to have reliability and validity if necessary)?
8. Was the same mode of data collection used for all subjects?
9. Was the length of the shortest prevalence period for the parameter of interest appropriate?
10. Were the numerator(s) and denominator(s) for the parameter of interest appropriate?

**Supplementary figure 1.** Funnel plots for meta-analysis for prevalence of a) hypertension b) elevated blood pressure and c) combined elevated blood pressure and hypertension.

**A)**
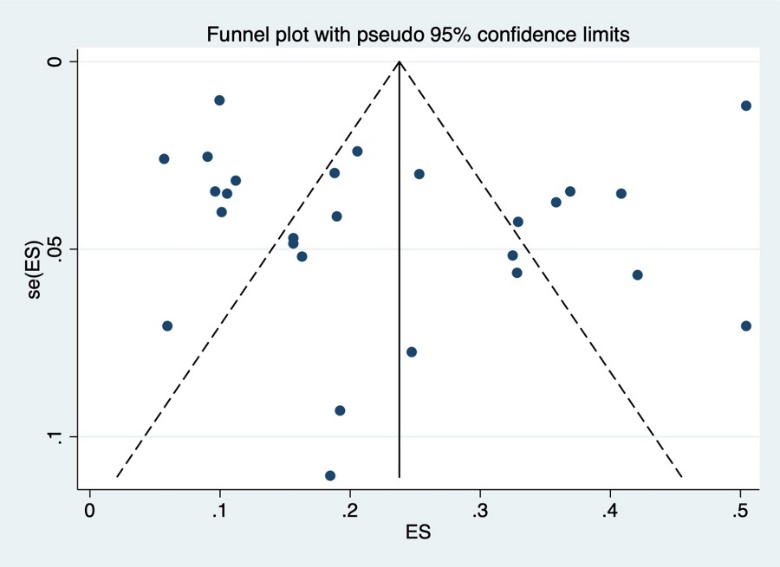

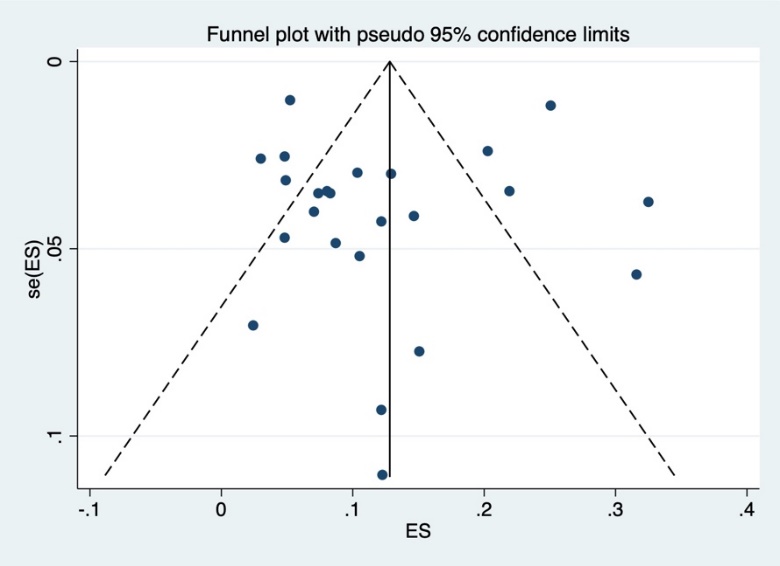

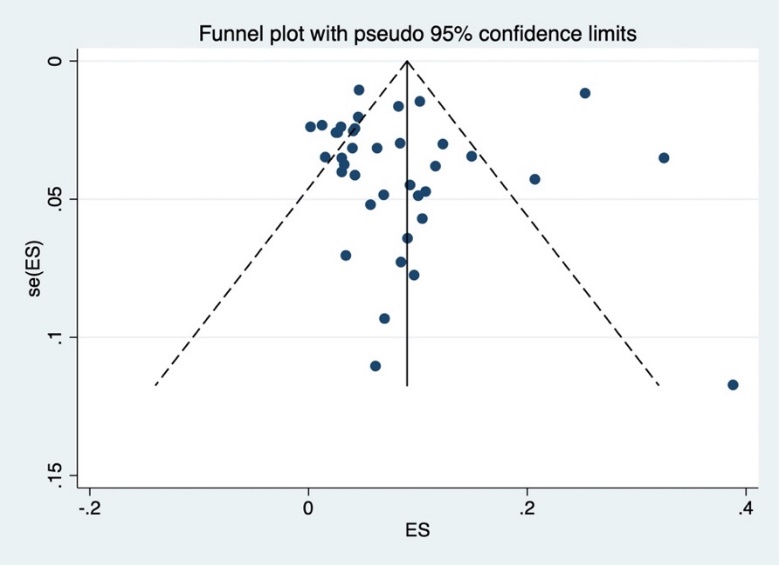
 **B) C)**

**Supplementary figure 2**. Funnel plots for hypertension meta-analysis by subgroup: Africa region


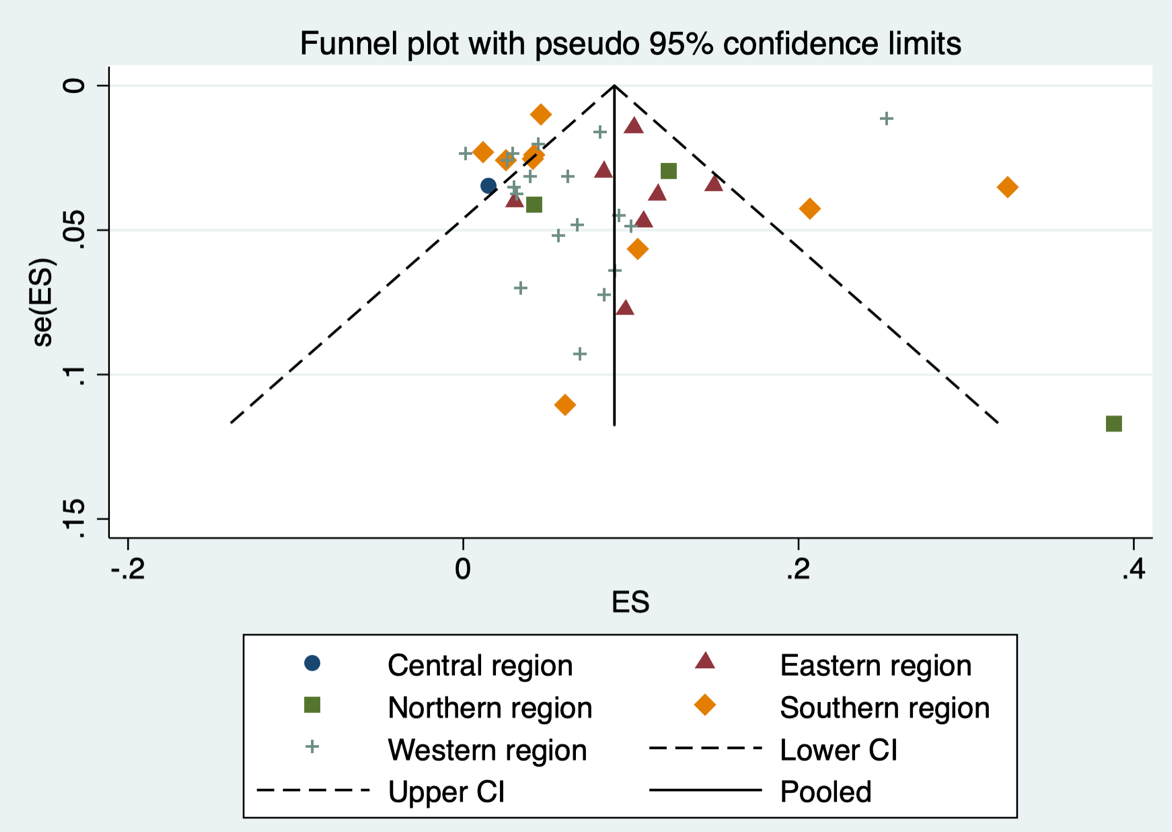


**Supplementary figure 3**. Funnel plots for hypertension meta-analysis by subgroup: Geographical setting


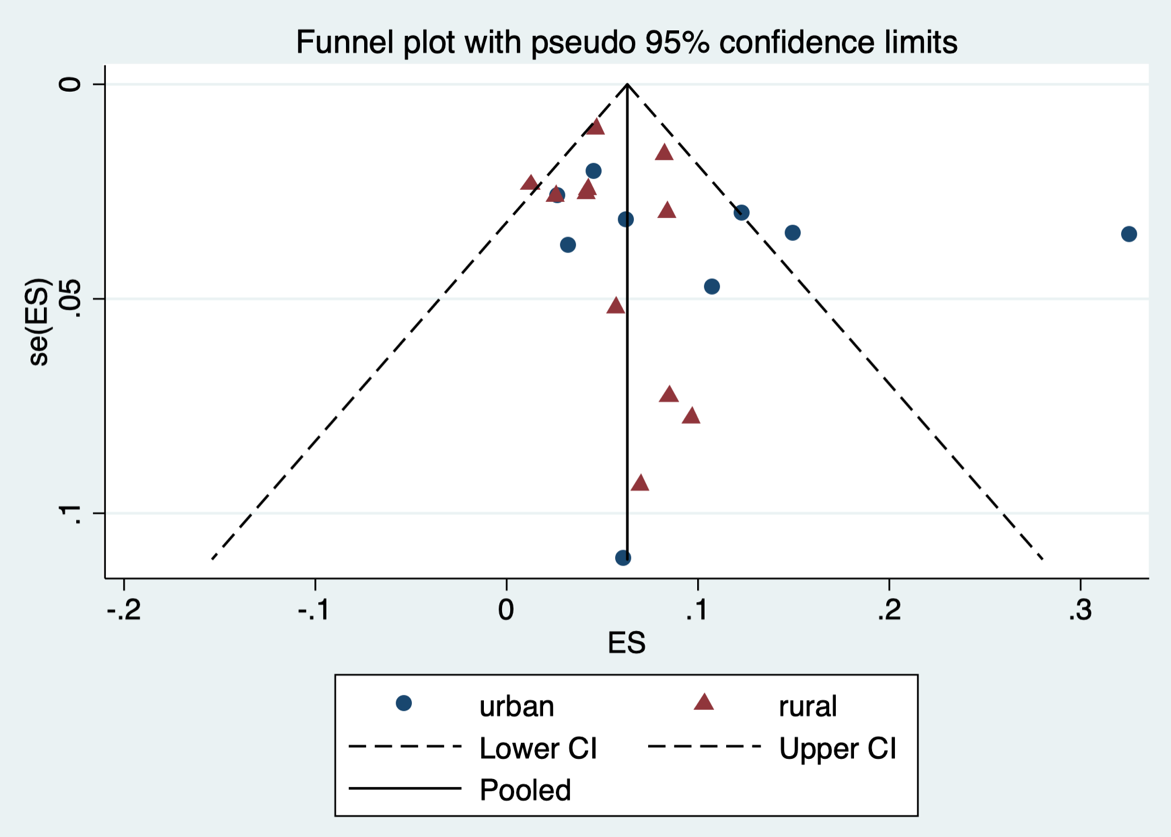


**Supplementary figure 4**. Funnel plots for hypertension meta-analysis by subgroup: Timing of data collection


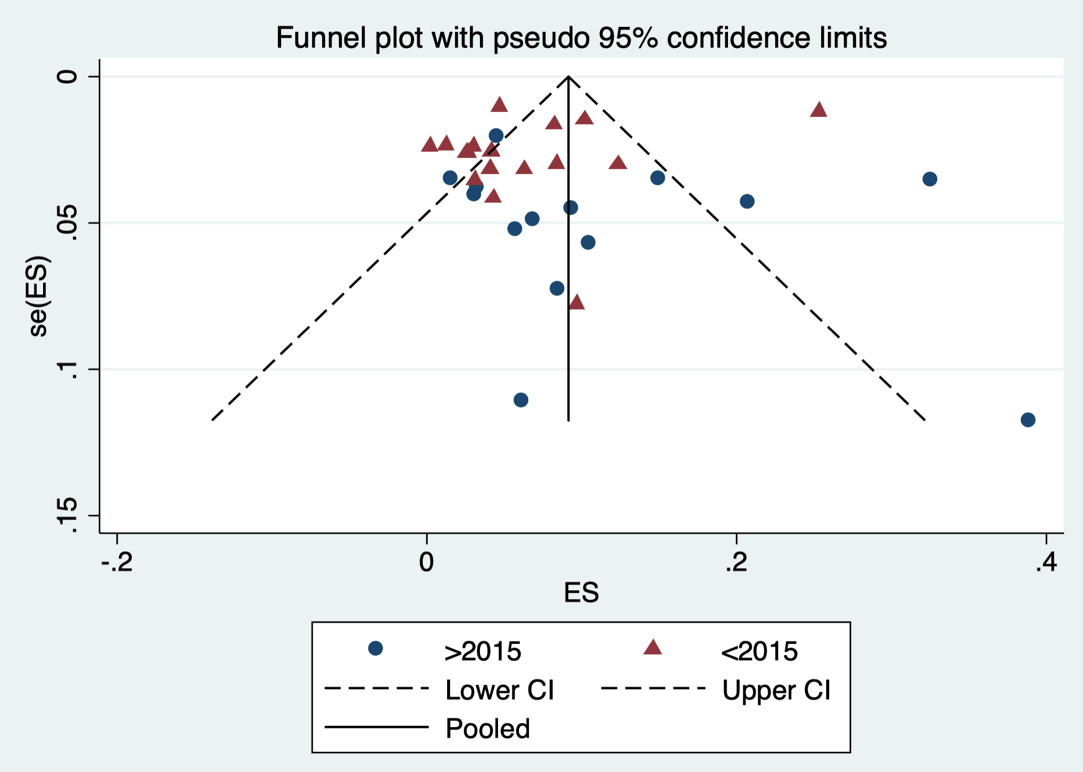


**Supplementary figure 5**. Funnel plots for hypertension meta-analysis by subgroup: Age group

**
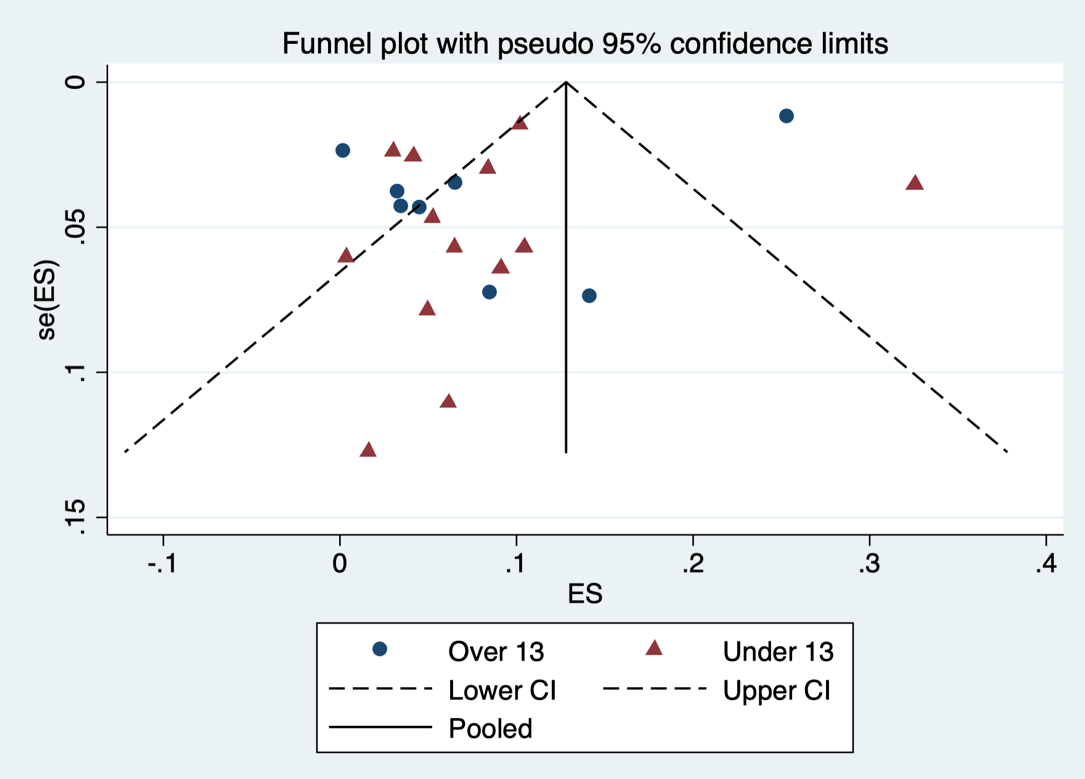
**

**Supplementary figure 6**. Funnel plots for hypertension meta-analysis by subgroup: Sex


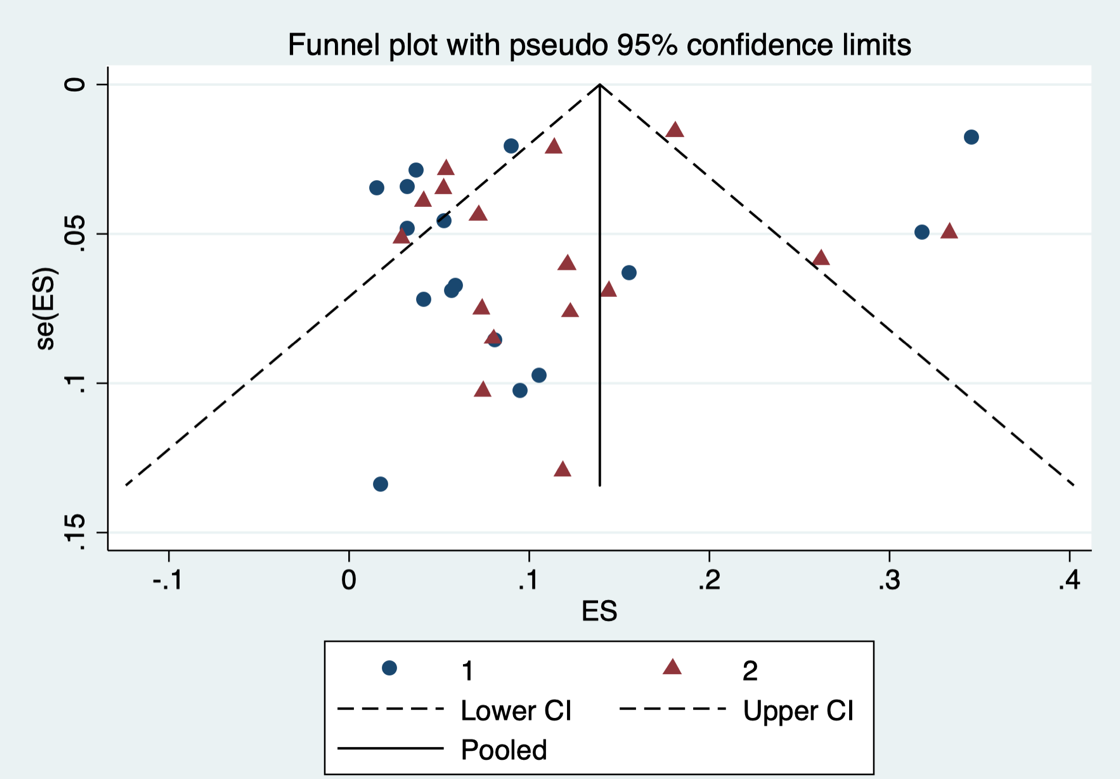


**Supplementary figure 7**. Funnel plots for hypertension meta-analysis by subgroup: BMI category


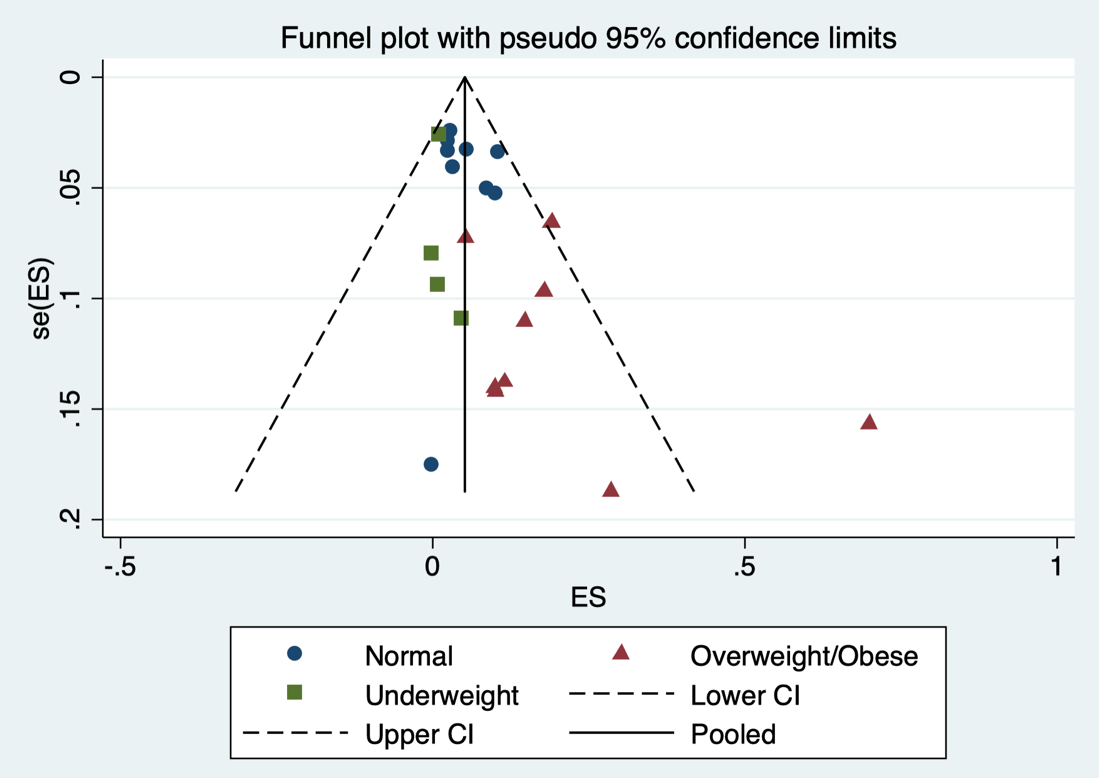


**Supplementary figure 8**. Funnel plots for hypertension meta-analysis by subgroup: BP methodology


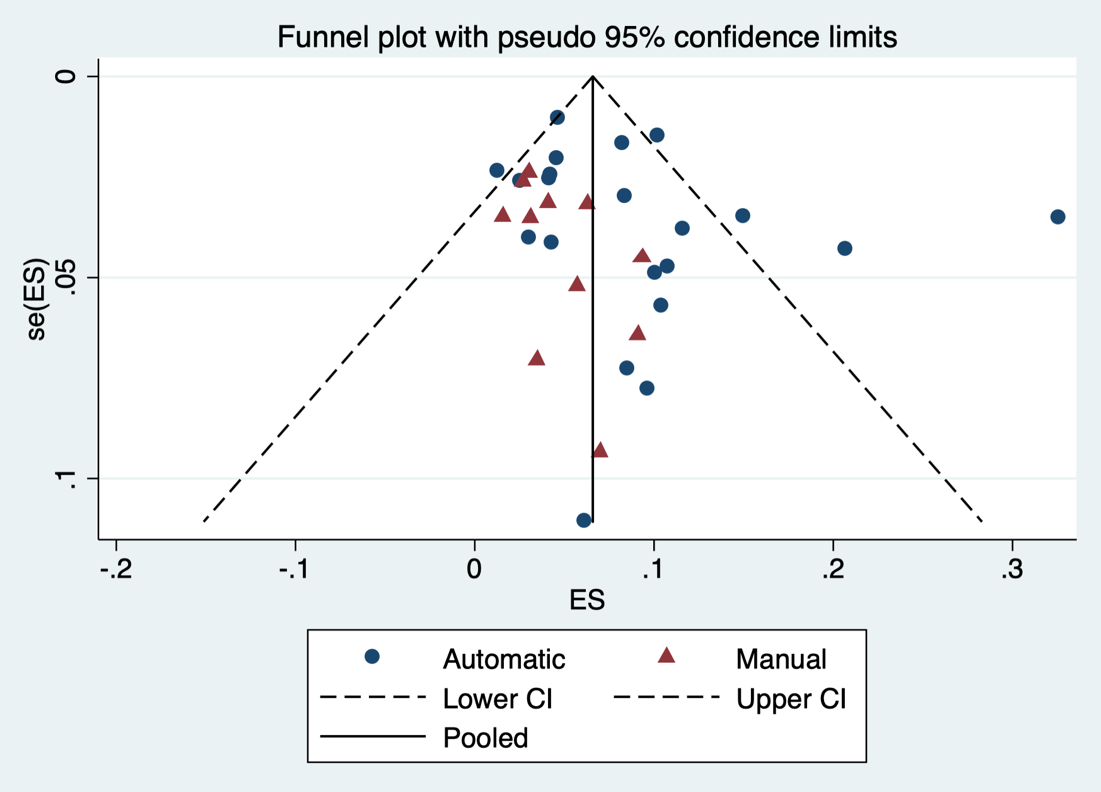


**Supplementary figure 9**. Funnel plots for hypertension meta-analysis by subgroup: Number of measurement occasions


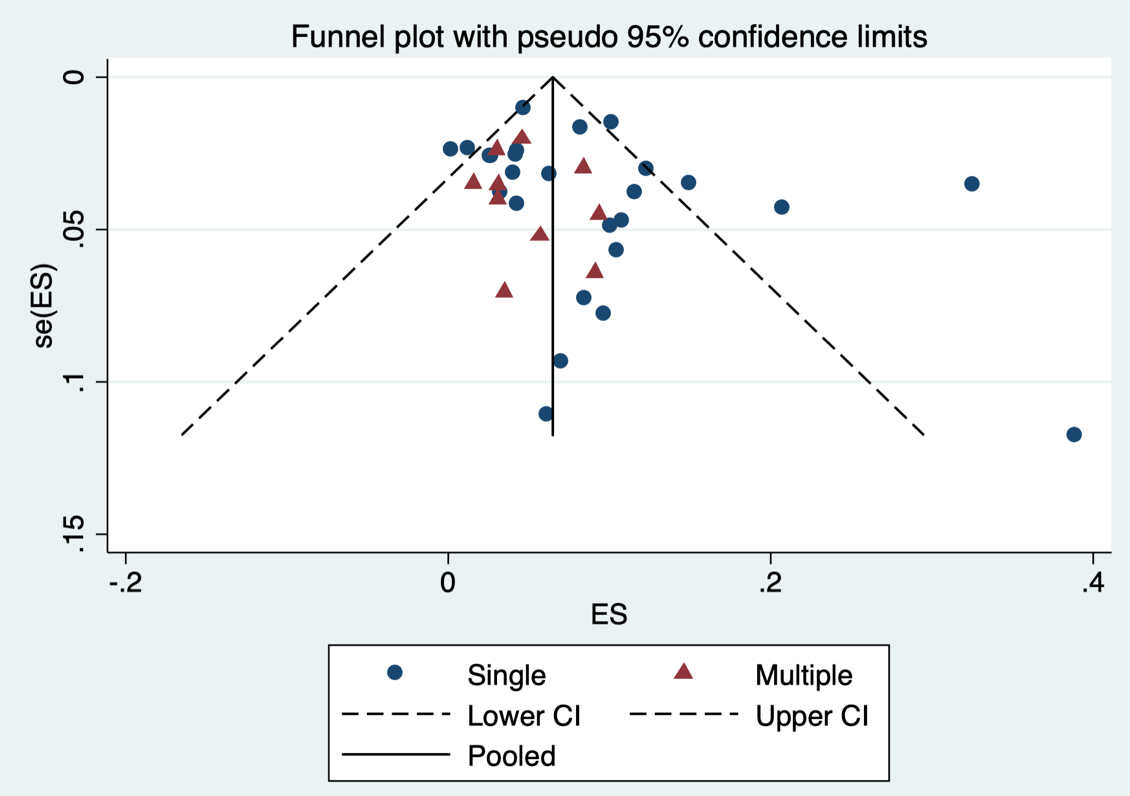


**Supplementary figure 10**. Funnel plots for hypertension meta-analysis by subgroup: Standards used for classification of HTN


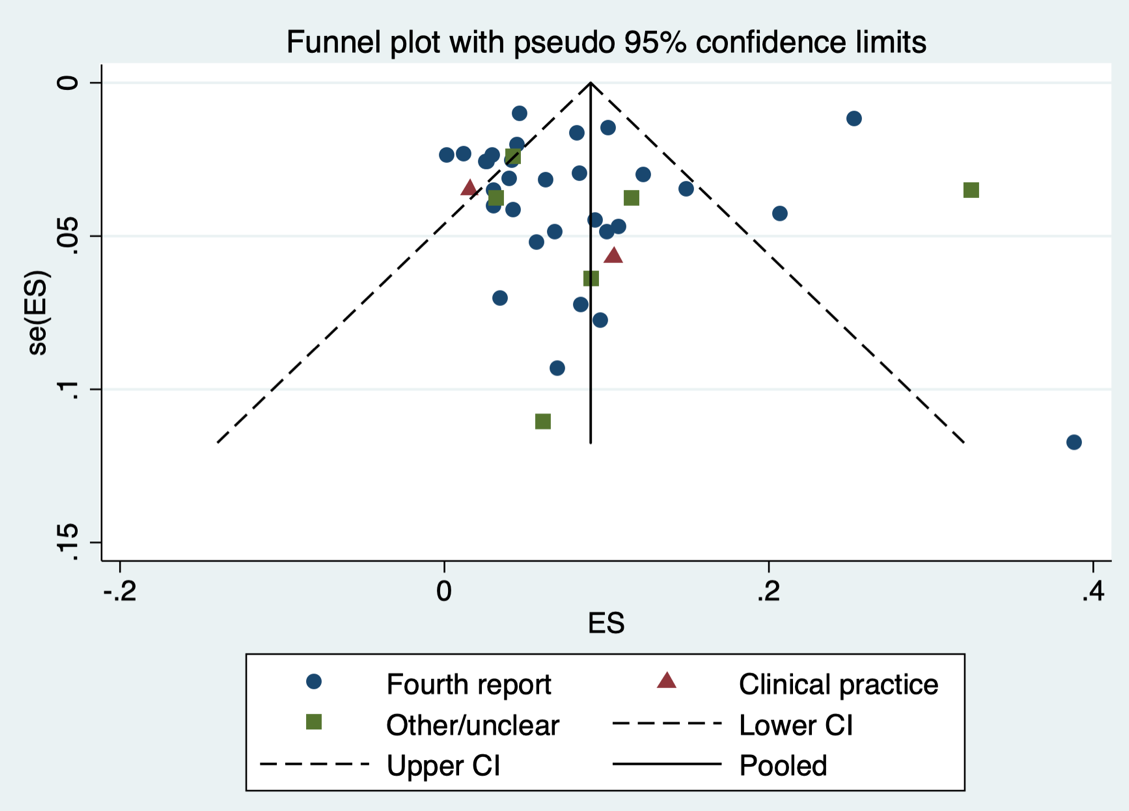


**Supplementary figure 11**. Funnel plots for hypertension meta-analysis by subgroup: Sample size


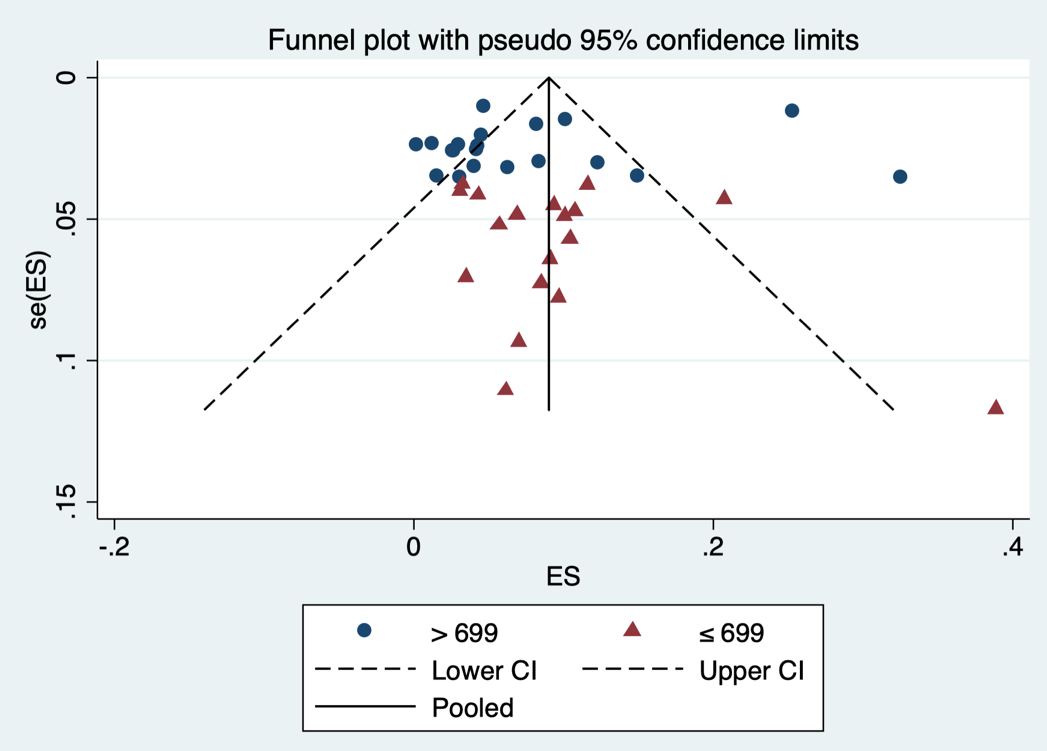


**Supplementary figure 12**. Funnel plots for hypertension meta-analysis by subgroup: Risk of bias score


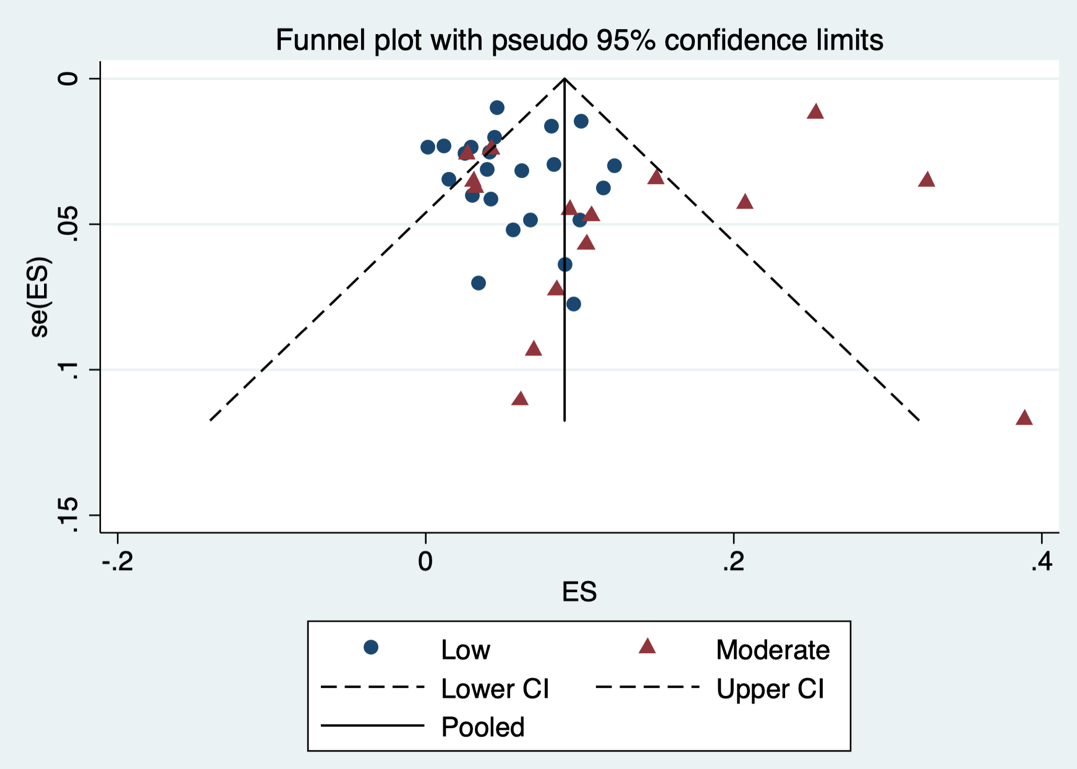


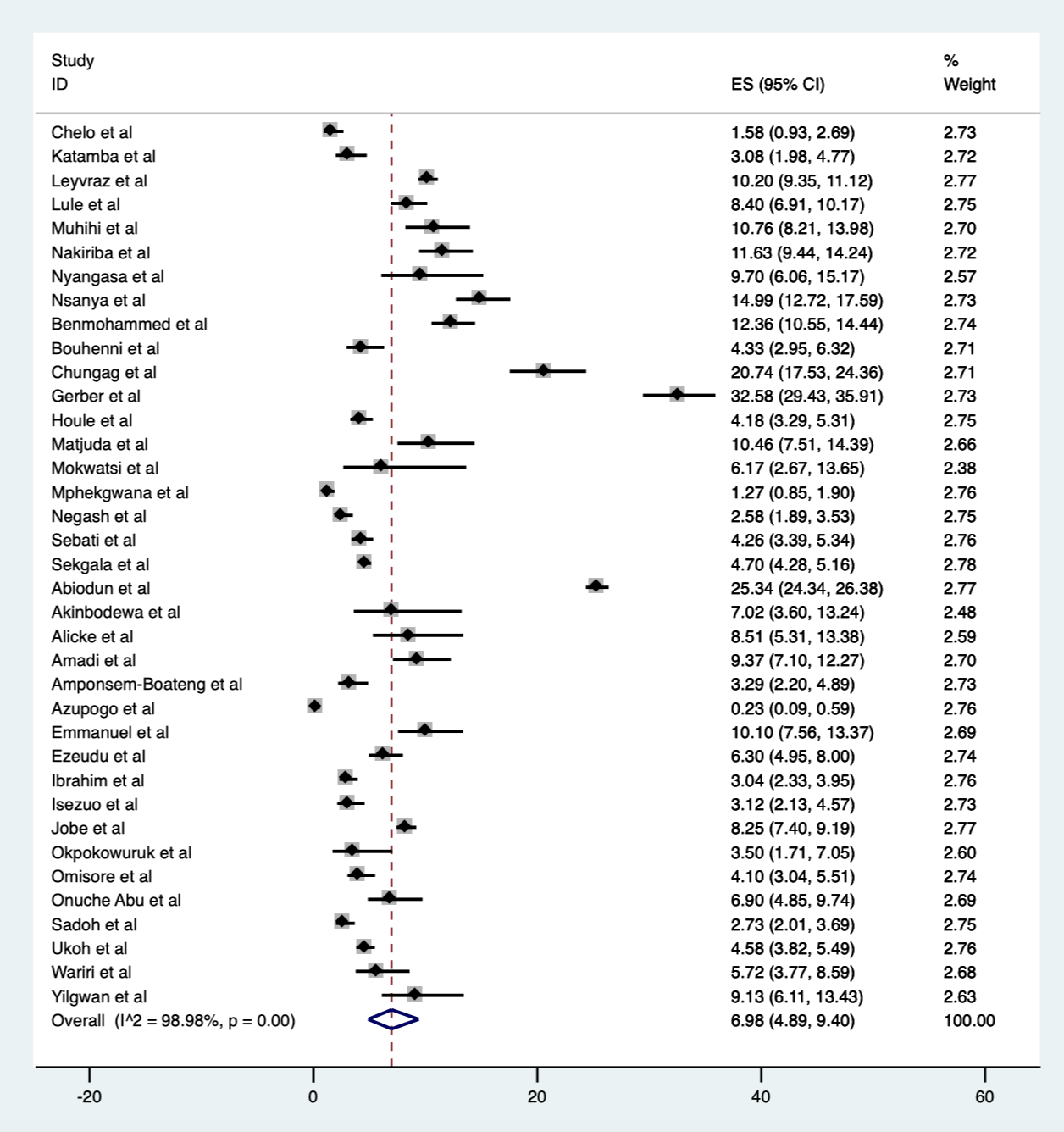
**Supplementary figure 13.** Meta-analysis for hypertension prevalence excluding study (ref: 44) not reporting, or adjusting for lack of, multiple measures.
